# Associations between 24-h movement behaviors and indicators of mental health and well-being across the lifespan: a systematic review

**DOI:** 10.1186/s44167-024-00048-6

**Published:** 2024-03-14

**Authors:** Claire I. Groves, Christopher Huong, Carah D. Porter, Bryce Summerville, Isabella Swafford, Braden Witham, Matt Hayward, Matthew Y. W. Kwan, Denver M. Y. Brown

**Affiliations:** 1 Department of Psychology, The University of Texas at San Antonio, San Antonio, TX 78249 USA; 2 Dolph Briscoe Jr Library, University of Texas Health San Antonio, San Antonio, TX 78229 USA; 3 Department of Child and Youth Studies, Brock University, St. Catharines, ON L2S 3A1 Canada

**Keywords:** Mental disorders, Physical activity, Screen time, Sedentary behavior, Sleep

## Abstract

Researchers have adopted a variety of analytical techniques to examine the collective influence of 24-h movement behaviors (i.e., physical activity, sedentary behaviors, sleep) on mental health, but efforts to synthesize this growing body of literature have been limited to studies of children and youth. This systematic review investigated how combinations of 24-h movement behaviors relate to indicators of mental ill-being and well-being across the lifespan. A systematic search of MEDLINE, PsycINFO, Embase, and SPORTDiscus was conducted. Studies were included if they reported all three movement behaviors; an indicator of mental ill-being or well-being; and were published in English after January 2009. Samples of both clinical and non-clinical populations were included. A total of 73 studies (*n* = 58 cross-sectional; *n* = 15 longitudinal) met our inclusion criteria, of which 47 investigated children/youth and 26 investigated adults. Seven analytical approaches were used: guideline adherence (total and specific combinations), movement compositions, isotemporal substitution, profile/cluster analyses, the Goldilocks method and rest-activity rhythmicity. More associations were reported for indicators of mental ill-being (*n* = 127 for children/youth; *n* = 53 for adults) than well-being (*n* = 54 for children/youth; *n* = 26 for adults). Across the lifespan, favorable benefits were most consistently observed for indicators of mental well-being and ill-being when all three components of the 24-h movement guidelines were met. Movement compositions were more often associated with indicators of mental health for children and youth than adults. Beneficial associations were consistently observed for indicators of mental health when sedentary behavior was replaced with sleep or physical activity. Other analytic approaches indicated that engaging in healthier and more consistent patterns of movement behaviors (emphasizing adequate sleep, maximizing physical activity, minimizing sedentary behaviors) were associated with better mental health. Favorable associations were reported less often in longitudinal studies. Collectively, these findings provide further support for adopting an integrative whole day approach to promote mental well-being and prevent and manage mental ill-being over the status quo of focusing on these behaviors in isolation. This literature, however, is still emerging—for adults in particular—and more longitudinal work is required to make stronger inferences.

**Supplementary Information:**

The online version contains supplementary material available at 10.1186/s44167-024-00048-6.

## Introduction

Poor mental health and high rates of mental illness are major concerns across the lifespan in society today. Findings from the 2019 Global Burden of Disease Study estimated that roughly one in eight adults have a mental disorder [1], whereas the prevalence is slightly lower among children and adolescents at one in twelve individuals [2]. Depression and anxiety represent the most common mental health problems, and rates of these disorders have risen further since the onset of the COVID-19 pandemic [3]. Beyond clinically diagnosed mental disorders, recent evidence has also found overall declines in mental health and well-being across the lifespan [4, 5]. Collectively, the impacts of poor mental health and mental illness are felt at many levels in our society—whether it be at the individual, family, school or workplace—and place a considerable burden on health care systems and the economy [6, 7]. The economic burden of mental disorders is only expected to rise, with projections estimating an economic burden of $6 trillion by 2030 [8]. Such evidence underscores the need to better understand the etiology of mental well-being and mental disorders.

Historically, mental health research has predominantly focused on psychopathology and distress, adopting a pathogenic perspective based on the medical model of disease. This model views mental health as merely the absence of mental illness, such as depression or anxiety. However, recent developments have brought forth the dual continua model of mental illness and health [9]. This model recognizes that mental illness and mental health are related but distinct dimensions. Keyes' [9] seminal research provided robust evidence for this model, suggesting that individuals range from languishing to flourishing on a mental health continuum, while also existing on a parallel continuum of mental illness, characterized by the presence or absence of psychopathological symptoms. The dual continua model thus offers a more holistic understanding of mental health, promoting a refined view that covers the entire spectrum from dysfunction to optimal mental well-being. It has been proposed that widespread and systematic adoption of the dual-continua model would inspire significant reform to the mental health care system, which may better prepare systems for the overwhelming burden of mental illness [10]. It is therefore imperative that we identify cost-effective strategies that can not only assist in the prevention and/or management of mental ill-being, but also promote mental well-being.

Mental health promotion efforts targeting modifiable lifestyle behaviors are receiving growing interest as a potential low-cost alternative to traditional psychotherapy and pharmacological interventions [11, 12]. Among these behaviors there has been considerable recent attention paid to how much we move, remain stationary, and sleep over the course of a 24-h period. While there is no consensus terminology for referring to these behaviors collectively [13], they are most commonly referred to as 24-h movement behaviors, the 24-h activity cycle, physical behaviors, time-use behaviors or time-use activity behaviors. We will use the term 24-h movement behaviors for the purpose of this review. Prior to the emergence of the notion that all movement behaviors we engage in over the course of a whole day are important for health [14], researchers generally examined physical activity, sedentary behaviors and sleep independently. Reviews of observational studies have generally shown that engaging in greater amounts of moderate-to-vigorous intensity physical activity [12, 15–22] and lower amounts of sedentary behavior, including recreational screen time [23–29], are associated with greater mental well-being and reduced risk of mental ill-being among children and youth as well as adults. It should be noted that context has been acknowledged to play an influential role in these relationships [20, 30], although existing literature has arguably placed a greater emphasis on duration, volume or intensity-based metrics. Studies investigating sleep duration, on the other hand, have suggested an optimal range may exist wherein too much or too little sleep is associated with poorer scores on indicators of mental health and greater risk of mental disorders [12, 31–33]. This siloed approach neglected the fact that movement behaviors are co-dependent (i.e., time spent engaging in one behavior reduces time available for other behaviors) and interact to influence health [34]. This was the impetus for development of 24-Hour Movement Guidelines, which were first released in Canada in 2016 with recommendations specific to children and youth [35]. Several countries have since adopted 24-Hour Movement Guidelines (e.g., [36–38]), and these recommendations have been tailored for other segments of the population (e.g., early childhood, adults, older adults) [39, 40]. Consequently, the release of these integrative guidelines has sparked an emergence of studies seeking to answer different research questions regarding how combinations of 24-h movement behaviors relate to a variety of health outcomes [41, 42].

To date, two systematic reviews and one scoping review have investigated associations between 24-h movement behaviors and indicators of mental health [43–45]. These reviews focused strictly on children and youth, which is likely attributable to the 24-Hour Movement Guidelines for Children and Youth being published first in 2016 [35]. Nevertheless, evidence from the systematic reviews generally suggested that adhering to an increasing number of the 24-h movement guidelines or all three guidelines (compared to none) were associated with favorable benefits for several indicators of mental health, risk of depression and depressive symptoms in particular [44, 45]. It is important to acknowledge, however, that the studies included in the systematic reviews were largely cross-sectional and analyses tended to focus on guideline adherence as opposed to other approaches such as compositional data analysis techniques, which are particularly appropriate for analyzing whole day time-use data given the approach takes the co-dependent nature of these behaviors into consideration, ultimately reducing bias in estimates [46]. The more recent scoping review by de Lannoy et al. [43] captured many more articles (*n* = 42) that examined associations between 24-h movement behaviors and mental health, including eight studies using compositional data analysis techniques. Among the studies included in their review, 21/27 (78%) and 23/27 (85%) studies that investigated indicators of mental well-being and ill-being [12], respectively, reported favorable associations with 24-h movement behaviors when assessed collectively. However, findings across analytic approaches were amalgamated, which fails to convey important insights from the different research questions examined. Beyond the literature focused on children and youth, new evidence is emerging rapidly for adults, but these findings have yet to be synthesized to gain a more comprehensive understanding of how combinations of 24-h movement behaviors relate to mental health during adulthood. If the favorable associations observed among young people extend across the broader lifespan, such findings would further support the importance of adopting the integrative whole day approach to health over the status quo of examining these behaviors in isolation. Therefore, the purpose of this study was to conduct a systematic review to examine how combinations of 24-h movement behaviors relate to indicators of mental ill-being and well-being across the lifespan.

## Methods

### Protocol and registration

This review was prospectively registered with the International Prospective Register of Systematic Reviews (PROSPERO; submitted July 13, 2022; ID: CRD42022345672). The Preferred Reporting Items for Systematic Reviews and Meta-Analyses (PRISMA) guidelines were followed [47], and items are reported using the PRISMA Checklist (see Additional file 1).

### Inclusion criteria

We included studies that met the following criteria: (a) measured all three movement behaviors (i.e., physical activity, sedentary/screen time, sleep duration); (b) assessed at least one emotional or psychological indicator of mental well-being (e.g., flourishing, life satisfaction, self-esteem) or ill-being (e.g., depressive symptoms, psychological distress, suicidal ideation); (c) examined the effect sizes of associations between 24-h movement behavior combinations with at least one mental health indicator (e.g., regression coefficients, odds ratios, risk ratios, etc.); (d) were published after 2009; and (e) were published in the English language. Mental health was operationalized as a multidimensional construct involving facets of either mental well-being or ill-being based on Keyes’ [9] dual continua model. Emotional and psychological indicators of mental health were focused on given their introspective and interconnected nature (e.g., emotional states can influence mental functioning related to thought patterns and behaviors, and vice versa). For measures of mental ill-being, these included psychiatric symptoms as well as disorders (e.g., depressive symptoms, risk of being diagnosed with depression). Measures of well-being involved a focus on positive attributes of mental health (e.g., flourishing, self-esteem) and emotions (e.g., affect, happiness). Items/scales that measured quality of life were included as an indicator of mental health if they assessed happiness or life satisfaction, which are considered aspects of mental well-being [48]. In contrast, measures of health-related quality of life (HRQoL) were excluded based on the premise that they assess health-related aspects of life quality beyond strictly mental health (e.g., physical, motor skills, social, etc.). The year 2009 was selected as the start date of our search given it preceded the first paper (to our knowledge) which acknowledged the co-dependence between movement behaviors by five years [49], and thus was expected to capture all studies that examined how combinations of 24-h movement behaviors relate to mental health. Studies were excluded if they (a) were not a peer-reviewed article (i.e., Masters thesis, PhD dissertations, conference abstracts); (b) assessed health-related quality of life or cognitive-based indicators of brain health; and (c) specified 24-h movement behaviors as the outcome as opposed to the exposure in the statistical model.

### Search strategy, data extraction, and data synthesis

An electronic search was conducted in the MEDLINE, PsycINFO, Embase, and SPORTDiscus databases in July 2022 and subsequently updated in February 2023 and August 2023. These databases were searched based on their relevance to the review topic and for consistency with previous reviews examining associations between 24-h movement behaviors and mental health among children and youth that also searched these databases. A manual search of the new non-indexed *Journal of Activity, Sedentary and Sleep Behaviors* was also performed given the relevance of its scope to the purpose of the present review. Search terms can be found in Additional file 2. The search strategy did not specify indicators of mental health as the language used to describe such measures tends to be heterogenous and some studies simply state “health outcomes”, which would have led to exclusion at the title and abstract stage when mental health may have been assessed. References were imported to the review management software Covidence (Evidence Partners, Ottawa, ON, Canada), where duplicates were removed and titles/abstracts reviewed by two independent reviewers for initial inclusion. After initial screening, full-texts were retrieved and independently examined for final inclusion. Any conflicts during each stage were resolved through discussion amongst the research team. References of included articles and relevant reviews were further searched to identify studies which may have been missed by the database searches. Data extraction was performed independently by two reviewers and a third reviewer examined the data for consensus, and included: (a) publication year; (b) demographic measures (e.g., age, sex, country); (c) sample size; (d) measure of movement behaviors; (e) measure(s) of mental health; (f) the statistical analysis employed; and (g) key findings.

Given the heterogeneity in the samples and statistical analysis techniques used to examine the relationships between 24-h movement behaviors and indicators of mental health, a quantitative synthesis (i.e., meta-analysis) was not conducted. Therefore, we proceeded with a narrative synthesis, and studies were reviewed and compared on a variety of characteristics including: age (children and youth, adults); study design (cross-sectional, longitudinal), indicator of mental health, and statistical analysis technique employed.

### Methodological quality and risk of bias assessment

All included studies used an observational design, therefore an adaptation of the National Institute of Health’s Quality Assessment Tool for Observational Cohort and Cross-sectional Studies (QATOCCS [50]) was used to assess the methodologic quality and validity of each study as well as their risk of bias. Study quality and risk of bias was assessed independently by two reviewers on the 14 criteria assessing clarity in reporting (e.g.., research question, population details), justification of methodological choices (e.g., reliability and validity of measurement tools, sample size), and use of best practices (e.g., repeated assessments, adjusting for confounders). Each study received a “yes”, “no”, or “other” response to each question to then be rated as “poor”, “fair”, or “good” based on these considerations as concerned with the exposure (i.e., 24-h movement behaviors) and outcomes of interest (i.e., indicators of mental health). The responses are intended to be used as a guide for assessing the quality and risk of bias rating, however, in line with previous work that has used ranges of scores to provide quantitative evaluations [51, 52], we considered studies with a score between ≤ 4 to be poor, 5 to 9 to be fair, and > 9 to be good.

## Results

### Included studies

**Fig. 1:**
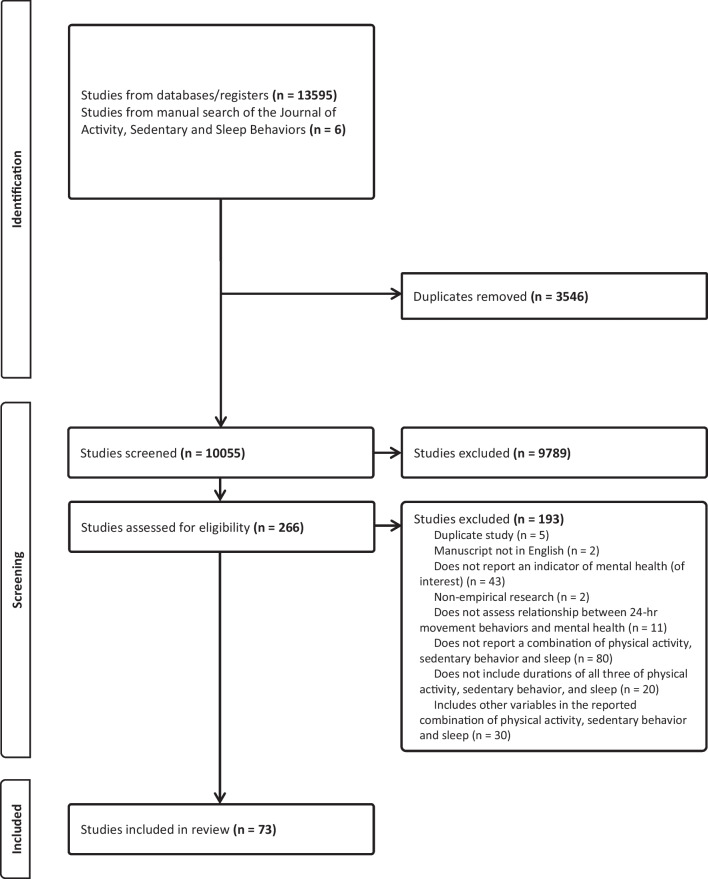
PRISMA flow diagram

The initial search identified 11,339 records, which was reduced to 8,749 after duplicates were removed. Our updated searches in February and August identified an additional 1,366 and 890 records, which was reduced to 731 and 569 records after duplicates were removed. A total of 6 articles were identified in our manual search of the *Journal of Activity, Sedentary and Sleep Behaviors.* In total, 10,055 records were identified across the searches. A team of seven reviewers screened the titles and abstracts for inclusion (two independent reviewers per study), resulting in 266 records that were moved to the full text review stage. Two independent reviewers then read and assessed the full text articles for inclusion, ultimately resulting in a total of 73 studies that met all inclusion criteria. At both screening stages, any disagreements were resolved through discussion and consensus among the screening team. A PRISMA flow diagram is presented in Fig. 1.

### Description of studies

Study details and outcomes are presented and briefly summarized in Table 1. Studies included were published between 2017 and 2023 with samples from 16 different countries: Canada (*n* = 22), United States (*n* = 15), China, including Hong Kong (*n* = 15), Australia: (*n* = 8), United Kingdom (*n* = 4), South Korea (*n* = 2), Spain (*n* = 2), Brazil (*n* = 2), Finland (*n* = 1), Japan (*n* = 2), New Zealand (*n* = 2), Singapore (*n* = 1), Sweden (*n* = 1), Switzerland (*n* = 1), and The Netherlands (*n* = 1), Belgium (*n* = 1). A total of 47 studies included samples of children and youth (414,542 total participants with a rangefrom 88 to 73,074 participants across studies, and ranging from 3 to 17 years of age), of which 37 used cross-sectional designs and 10 used longitudinal designs. For adults, a total of 26 studies were included in our review (183,163 participants with a range of 200 to 60,235 participants across studies, and ranging from 15 to 79 years of age), of which 21 used cross-sectional designs and five used longitudinal designs. The follow-up time ranged from 6-months to 22-years for prospective studies. A total of 65 studies included non-clinical samples (children and youth: *n* = 42; adults: *n* = 23) and 8 studies included clinical samples (children and youth: *n* = 5; adults: *n* = 3). The same datasets were used in multiple studies: two studies used the Application of integrateD Approaches to understanding Physical activity during the Transition to emerging adulthood (ADAPT) dataset [53, 54], two studies used a sample of children from two studies conducted in the United Kingdom [55, 56], two studies used the National Longitudinal Study of Adolescent to Adult Health (Add Health) [57, 58], three studies used the National Survey of Children’s Health (NSCH) [59–61], and the similarities of the study design and participant characteristics across three studies[62–64] suggested the same unnamed dataset was used. Associations between combinations of 24-h movement behaviors and indicators of mental health were assessed using seven different analytical approaches: total guideline adherence (i.e., adhering to none, one, two or all three of the guidelines), specific combinations of guideline adherence (i.e., adhering to none, physical activity, sleep, sedentary behavior/screen time, physical activity and sleep, sleep and sedentary behavior/screen time, physical activity and sedentary behavior/screen time, or all three guidelines concurrently), compositional data analysis, the Goldilocks approach (within a compositional data analysis framework, which aims to determine the optimal time-use composition for indicators of health), isotemporal substitution (traditional and compositional data analysis models), rest/activity rhythmicity, and latent profile or cluster-based analyses.

**Table 1:** Characteristics of included studies and main findings

Authors (year) and study design	Country	PA measure	SB measure	Sleep measure	Mental Health outcome	*N* and Statistical Analysis	Age in years: Mean and/or range	Sex/Gender	Main findings
Baillot et al., (2022) [101] Cross-sectional	Canada	Accelerometer (ActiCal—right hip) MVPA	Questionnaire (self-reported screen time use)	Questionnaire (average hours/day)	General mental health (self-rated)	10,515; Logistic regression	M = 45; Range = 18–79	49.64% female	Meeting 1* guideline was associated with significantly better odds of very good or excellent mental health among Obese 1 group; No associations observed for Normal Weight, Overweight and Obese 2 groups
Bang et al., (2020) [102] Cross-sectional	Canada	Accelerometer (Actical – right hip) MVPA	Questionnaire (weekly recreational ST; Parent-reported for ages 6–11 years, Child-reported for ages 12–17 years)	Questionnaire (average hours/day; Parent-reported for ages 5–11 years, Child-reported for ages 12–17 years)	Emotional difficulties (SDQ); General mental health (youth only)	4,250 (2773 children; 1477 youth); Logistic regression	Range = 5–17	48.7% Female (5–11 years); 48.8% Female (12–17 years)	Children: Meeting any number or specific combination of guidelines was not associated with less emotional difficulties compared to meeting none of the guidelines Youth: Meeting 2 + * (total) guidelines and the SL + PA* (combination) guidelines were associated with more favorable scores for emotional difficulties compared to meeting none of the guidelines Youth: Meeting any number or specific combination of guidelines was not associated with better mental health compared to meeting none of the guidelines
Blodgett et al., (2023) [103] Cross-sectional	United Kingdom	Accelerometer (activPAL—thigh) LPA and MVPA	Accelerometer (activPAL—thigh worn) SB	Accelerometer (activPAL—thigh worn) SL	Depression (anti-depressant medication prescription and visited doctor due to depressive symptoms)	4738; CoDA, including ISM	M = 46	52.3% female	CoDA: SL was associated with greater risk of depression, whereas LPA and MVPA were associated with lower risk of depression ISM: - + MVPA/-SL or SB or LPA: lower risk of depression - + LPA/-SL or SB: lower risk of depression - + SL/-MVPA or LPA or SB: greater risk of depression
Brown, Cairney, et al., (2021) [53] Cross-sectional	Canada	Questionnaire (weekly MVPA via IPAQ-SF)	Questionnaire (average daily time spent using devices during free time)	Questionnaire (average sleep duration)	Flourishing (FS); Self-Esteem (RSE); Resiliency (two-item measure)	1166; Latent profile analysis	M = 16	54% female	Sleep patterns were similar (adequate amount) across profiles so profiles were characterized by differences in ST & MVPA Flourishing: High MVPA/Low ST * > Low MVPA/Low ST > High MVPA/High ST & Low MVPA/High ST Self-Esteem: High MVPA/Low ST * > Low MVPA/Low ST & High MVPA/High ST & Low MVPA/High ST Resiliency: High MVPA/Low ST > Low MVPA/Low ST; High MVPA/Low ST = High MVPA/High ST; High MVPA/High ST > Low MVPA/Low ST; High MVPA/Low ST & Low MVPA/Low ST & High MVPA/High ST > Low MVPA/High ST
Brown, McPhee et al., (2021) [59] Cross-sectional	United States	Questionnaire (parent-reported days of physical activity per week with 60 or more minutes)	Questionnaire (parent-reported average daily hours of recreational screen time use)	Questionnaire (parent-reported hours of sleep most weeknights)	Depression and Anxiety (parent indicated whether child received diagnosis from doctor)	8554; Logistic regression	Children with Neuro-developmental disorders: 12	37.3% female	Meeting 1 or 2* guidelines was associated with the lowest odds of anxiety and depression compared to meeting none
Brown and Ronen (2021) [60] Cross-sectional	United States	Questionnaire (parent-reported days of physical activity per week with 60 or more minutes)	Questionnaire (parent-reported average daily hours of recreational screen time use)	Questionnaire (parent-reported hours of sleep most weeknights)	Depression and Anxiety (parent indicated whether child received diagnosis from doctor)	663 (Active epilepsy); 526 (Previous epilepsy); Logistic regression	Active epilepsy: 11; Previous epilepsy: 12; Range = 6–17	50.5% Female (Active Epilepsy); 37% Female (Previous Epilepsy)	Meeting any number of guidelines was not associated with reduced odds of anxiety compared to meeting none of the guidelines Meeting 1 or all 3* guidelines was associated with the lowest odds of depression compared to meeting none
Brown, Kwan, et al., (2021) [57] Cross-sectional and longitudinal	United States	Questionnaire (weekly MVPA frequency)	Questionnaire (average weekly hours of ST)	Questionnaire (average hours of nightly SL)	Depressive symptoms (CES-D)	6436; Latent profile analysis	M = 16	51.7% female	Sleep patterns were similar (adequate amount) across profiles, so profiles were characterized by differences in ST & MVPA Cross-sectional and longitudinal (1-year later): Depressive symptoms: High MVPA/low ST profile (profile 1)* < High MVPA/High ST = Low MVPA/Low ST = Low MVPA/high ST
Brown and Kwan (2021) [54] Cross-sectional	Canada	Questionnaire (weekly MVPA via IPAQ-SF)	Questionnaire (average daily time spent using devices during free time)	Questionnaire (average hours of nightly SL)	Flourishing (FS); Self-Esteem (RSE); Resiliency (two-item measure)	1118; ISM	M = 16	54.5% Female	Flourishing (> = 8 h sleep): - + MVPA/-SL or ST: more favorable scores Flourishing (< 8 h sleep): - + MVPA or SL/-SB: more favorable scores Self-Esteem: - + MVPA or SL/-SB: more favorable scores Resiliency: - + MVPA or SL/-SB: more favorable scores
Brown, Faulkner et al., (2022) [104] Cross-sectional	Canada	Questionnaire (weekly MVPA via IPAQ-SF)	Questionnaire (average hours of weekday recreational ST)	Questionnaire (average hours of nightly SL)	Psychological Distress (K10); Mental wellbeing (WEMWBS)	15,080; Latent profile analysis	M = 21	67.1% Female	Sleep patterns were similar (adequate amount) across profiles, so profiles were characterized by differences in ST & MVPA Psychological Distress: High MVPA/Low ST* < Very high MVPA/Low ST = Low MVPA/Low ST < High MVPA/High ST = Low MVPA/High ST Mental well-being: High MVPA/Low ST * = Very High MVPA/Low ST *; Low MVPA/Low ST = High MVPA/High ST > Low MVPA/High ST
Brown, Hill, et al., (2022) [85] Cross-sectional	Canada	Questionnaire (weekly MVPA via IPAQ-SF)	Questionnaire (average daily hours of recreational ST via International Sedentary Assessment Tool); Questionnaire (average daily sitting time—SB)	Questionnaire (average hours of nightly SL)	Suicidal ideation; Suicidal planning	17,633; Logistic regression with covariate balancing propensity score weighting	M = 22	67.1% female	Meeting the PA + SL, SB + SL or all 3* guidelines was associated with the lower odds of suicidal ideation compared to meeting none Meeting the PA + SL* guidelines was associated with the lower odds of suicidal planning compared to meeting none
Bu al., (2021) [62] Cross-sectional	China	Questionnaire (weekly MVPA via IPAQ-SF)	Questionnaire (daily SB via IPAQ-SF)	Questionnaire (sleep duration via PSQI)	Anxiety symptoms (Chinese version of SAS)	1846; Logistic regression	M = 21	64% Female	Meeting any number of guidelines less than all three was associated with greater odds of anxiety symptoms
Burns et al., (2020) [105] Cross-sectional	United States	Questionnaire (days of physical activity per week with 60 or more minutes; days per week of muscle strengthening activity)	Questionnaire (average daily hours of recreational ST)	Questionnaire (hours of school-night sleep)	Perceived loneliness; Prolonged sadness	1897; Logistic regression	M = 16	48.4% Female (weighted)	Meeting the PA + SL*, ST + SL, or all 3 guidelines was associated with lower odds of perceived loneliness compared to meeting none Meeting all PA + ST, ST + SL, or all 3* guidelines was associated with lower odds of prolonged sadness compared to meeting none
Cabanas-Sánchez et al., (2021) [79] Cross-sectional and longitudinal	Spain	Accelerometer (ActiGraph wGT9x—wrist worn) LPA and MVPA	Accelerometer (ActiGraph wGT9x—wrist worn) SB	Accelerometer (ActiGraph wGT9x—wrist worn) SL	Depressive symptoms (GDS-10); Loneliness (three-item loneliness scale); Happiness (Cantril Ladder of Life Scale); Global mental health (12-item Short Form Health Survey)	2,489 (cross-sectional); 1,679 (prospective); CoDA, including ISM	Cross-sectional: M = 72; Longitudinal = 71	53.07% Female (cross-sectional); 51.70% Female (prospective)	Cross-sectional: CoDA: The 24-h movement composition was associated with depressive symptoms and happiness, and time spent in MVPA (relative to other behaviors) was associated with more favorable scores for both outcomes. No association was observed for the 24-h composition with general mental health or loneliness, although MVPA was associated with more favorable scores for loneliness (relative to other behaviors) ISM: - + MVPA/-SL or SB: lower depression symptoms - + SL or SB/-LPA: lower depression symptoms - + LPA or SL/-SB: better general mental health - + MVPA/- SB, LPA or SL: lower loneliness - + MVPA/- SB, LPA or SL: higher happiness Longitudinal: CoDA: The 24-h movement composition was not associated with depressive symptoms, loneliness, happiness or general mental health, although time spent in SL (relative to other behaviors) was associated with less favorable scores for depressive symptoms and time spent in MVPA was associated with more favorable scores for general mental health ISM: - + SB/-SL: lower depression symptoms ISM: - + MVPA/- SB, LPA or SL: better general mental health
Cao et al., (2020) [106] Cross-sectional	China	Questionnaire (days of physical activity per week with 60 or more minutes)	Questionnaire (average daily time spent using screen-based devices)	Questionnaire (typical hours of nightly sleep)	Depressive symptoms (CESD)	4178; Cluster analysis	M = 14	53.4% Female	Depressive symptoms: Active = High SL < High ST = Low MVPA/Low SL
Carson et al., (2019) [67] Cross-sectional	Canada	Accelerometer (Actigraph GT3X-BT – non-dominant wrist) LPA and MVPA	Questionnaire (parent-reported recreational ST)	Accelerometers (Actigraph GT3X-BT—non-dominant wrist)	Behavioral and emotional problems (parent report; CBCL)	539; Linear regression	M = 3	47.9% Females (outcome data present); 49.9% Female (exposure data present)	Meeting any number of guidelines was not associated with differences in total problems or internalizing problems compared to meeting all three guidelines Compared to meeting the PA and ST guidelines, meeting none of the guidelines was associated with higher total problems. Compared to meeting the ST and SL* guidelines, meeting none was associated with higher total problems and internalizing problems
Chao et al., (2022) [63] Cross-sectional	China	Questionnaire (weekly MVPA and walking via IPAQ-SF) LPA & MVPA	Questionnaire (average daily sitting time via IPAQ-SF) SB	Questionnaire (typical hours of nightly sleep)	Anxiety symptoms (SAS)	1475; CoDA, including ISM	M = 21	68.0% Female	CoDA: The 24-h movement composition was significantly associated with anxiety ISM (5–15 min): - + LPA/-SL: higher anxiety symptoms - + MVPA/-SL: higher anxiety symptoms - + LPA/-SB: higher anxiety symptoms - + MVPA/-SB: higher anxiety symptoms - + MVPA/-LPA: lower anxiety symptoms - + SL/-MVPA: lower anxiety symptoms
Chong et al., (2021) [78] Cross-sectional and longitudinal	Australia	Accelerometer (GENEActiv—wrist-worn) LPA and MVPA	1) Accelerometer (GENEActiv—wrist-worn) SB 2) Questionnaire (average time spent engaging in sedentary and screen-based activities on weekdays and weekends during a typical week) ST	Accelerometer (GENEActiv—wrist-worn) Sleep	Emotional problems, total difficulties (SDQ); Psychological Distress (K10)	127 (cross-sectional); 88 (longitudinal); CoDA	Cross-sectional: M = 12, Range = 10–12; Longitudinal: M = 13, Range = 11–13	57.5% Female (cross-sectional); 59% Female (longitudinal)	Cross-Sectional: CoDA: The 24-h movement composition was significantly associated with emotional problems and total difficulties, but not psychological distress. Relative to other behaviors, SL was associated with lower emotional problems scores, whereas SB and LPA were associated with higher scores; SL was associated with lower total difficulties scores, whereas LPA and ST were associated with higher scores; ST was associated with higher psychological distress scores Prospective (1 year): CoDA: No association between 24-h movement composition and emotional problems, total difficulties, or psychological distress
Christian et al., (2022) [107] Cross-sectional	Australia	Accelerometer (ActiGraph GT3X +—hip worn)	Questionnaire (parent-reported hours per day of recreational ST)	Questionnaire (parent-reported average daily sleep)	Emotional problems, total difficulties (SDQ)	1363; Linear regression	M = 3	47.98% female	For boys (but not girls), meeting the PA + SL, SB + SL*, or all 3 guidelines was associated with lower total difficulties compared to meeting none of the guidelines Meeting any combination of the guidelines compared to meeting none was not associated with more favorable scores for emotional problems for boys and girls
Colley et al., (2018) [108] Cross-sectional	Canada	Accelerometer (ActiCal—right hip worn) LPA and MVPA	Accelerometer (ActiCal—right hip worn) Sedentary time	Questionnaire (typical hours of daily sleep)	General mental health	10,621; ISM	M = 45; Range = 18–79	52.2% Female	- + LPA or SL/-SB: better mental health
Curtis et al., (2020) [109] Cross-sectional	Australia	Accelerometer (GENEActiv—wrist-worn) LPA and MVPA	Accelerometer (GENEActiv—wrist-worn) SB	Accelerometer (GENEActiv—wrist-worn) Sleep	Depression & Anxiety (DASS-21)	430; CoDA, including ISM	M = 41	74% Female	The 24-h movement composition was not associated with depression or anxiety
Curtis et al., (2023) [110] Cross-sectional	Australia	Accelerometer (Fitbit Charge 3—wrist) LPA and MVPA	Accelerometer (Fitbit Charge 3 -wrist) SB	Accelerometer (Fitbit Charge 3 -wrist) SL	Depression & Anxiety (DASS-21)	322; ISM	M = 40.4 (5.9)	58.1% Female	ISM(15 min) anxiety: - + LPA/-SL: lower anxiety scores - + LPA/-SB: lower anxiety scores ISM (15 min) depression: - + LPA/-SL: lower depression scores - + LPA/-SB: lower depression scores
de Faria et al., (2022) [111] Cross-sectional	Brazil	Accelerometer (ActiGraph wGT3x—right hip) LPA and MVPA	Accelerometer (ActiGraph wGT3x—right hip) SB	Accelerometer (ActiGraph wGT3x—right hip) SL	Depression/anxiety (GHQ-12)	217; CoDA, including ISM	M = 16; Range = 15–17	49.3% Female	CoDA: The 24-h movement composition was significantly associated with depression/anxiety. Relative to other behaviors, time spent in SB was associated with higher depression/anxiety, whereas LPA associated with lower depression/anxiety ISM (10 + min): - + LPA/-SB: lower depression/anxiety - + MVPA/-LPA: higher depression/anxiety
Del Pozo et al., (2020) [112] Cross-sectional	United States	Accelerometer (ActiGraph AM-7164—hip worn) LPA and MVPA	Accelerometer (ActiGraph AM-7164—hip worn) SB	Questionnaire (average hours of daily sleep)	Depressive symptoms (PHQ-9)	3233; CoDA, including ISM	M = 47	52.1% Female	CoDA: Time spent in SB (relative to other behaviors) was associated with greater depressive symptoms ISM: - + MVPA or SL/-SB: lower depressive symptoms
Dumuid et al., (2022) [82] Cross-sectional	Australia	Accelerometer (GENEActiv—wrist worn) LPA and MVPA	Accelerometer (GENEActiv—wrist worn) SB	Accelerometer (GENEActiv—wrist worn) SL	Life satisfaction (BMDSLSS); Psychosocial Quality of Life (PedsQL 4.0); Total difficulties (SDQ); Depression (MFQ-Short)	1182; CoDA, including Goldilocks method	M = 12 (0.4)	49% Female	The 24-h movement composition was significantly associated with life satisfaction, psychosocial QoL, depression, and total difficulties Goldilocks Day for each outcome: Life satisfaction: SL = 11.4 h; SB = 7.5 h; LPA = 2.9 h; MVPA = 2.2 h Psychosocial QoL: SL = 9.7 h; SB = 10.5 h; LPA = 1.7 h; MVPA = 2.1 h Depression: SL = 11.4 h; SB = 8.4 h; LPA = 2.3 h; MVPA = 1.9 h Total difficulties: SL = 11.4 h; SB = 7.3 h; LPA = 3.0 h; MVPA = 2.2 h
Duncan et al., (2022) [113] Longitudinal	Canada	Questionnaire (weekly MVPA)	Questionnaire (average hours of recreational ST per day) ST	Questionnaire (average hours of sleep per day) SL	Depressive symptoms (CESD-R); Anxiety symptoms (GAD-7); Flourishing (FS); Emotional dysregulation (DERS)	2645; ISM	Grade = 9–12	64.4% Female	ISM (10 min): Depressive symptoms: - + MVPA/-SL: greater depressive symptoms - + MVPA or SL/-ST: lower depressive symptoms Anxiety symptoms: - + MVPA/-SL: greater anxiety symptoms - + SL/-ST: lower anxiety symptoms Emotional Dysregulation: - + MVPA/-SL: greater emotional dysregulation - + MVPA or SL/-ST: lower emotional dysregulation Flourishing: - + MVPA or SL/-ST: greater flourishing
Fairclough et al., (2021) [55] Cross-sectional	United Kingdom	Accelerometer (ActiGraph wGT9x—non-dominant wrist) LPA and MVPA	Accelerometer (ActiGraph wGT9x—non-dominant wrist) SB	Accelerometer (ActiGraph wGT9x—non-dominant wrist) SL	Self-Esteem (RSE); Depressive symptoms (MFQ); Total difficulties (SDQ)	359; CoDA, including ISM	Primary school: M = 10.4 (0.7 SD) Secondary school: M = 12.0 (0.3 SD) Full sample: M = 11.5 (1.4 SD)	49.0% Female (Primary school); 53.0% Female (Secondary school); 50% Female (full sample)	Full Sample: CoDA: The 24-h movement composition was associated with internalizing problems, but not depression, self-esteem, or total difficulties. Relative to other behaviors, SB was associated with greater internalizing problems ISM (5–20 min): - + SB/-SL or MVPA: higher internalizing problems Primary school sample: CoDA: The 24-h movement composition was not associated with depression, self-esteem, total difficulties or internalizing problems Secondary school sample: The 24-h movement composition was not associated with any of the mental health outcomes
Fairclough et al., (2023) [56] Cross-sectional	United Kingdom	Accelerometer (ActiGraph GT9X—non-dominant wrist)	Accelerometer (ActiGraph GT9X—non-dominant wrist)	Accelerometer (ActiGraph GT9X—non-dominant wrist) SL	Total difficulties, internalizing problems (SDQ)	301; CoDA, including Goldilocks method	M = 11.1 (1.6)	60.13% Female	The average time-use composition was significantly associated with total difficulties (mental health) but not internalizing problems. ST was positively associated with overall total difficulties, whereas SL was negatively associated with total difficulties Inter-daily stability was negatively associated (less variability) with total difficulties (mental health) but not internalizing problems Intra-daily variability was not significantly associated with overall mental health or internalizing problems The most favorable composition: Total difficulties: SL = 10 h, ST = 6.5 h, LPA = 6.9 h, and MPA and VPA = 43 min
Feng et al., (2022) [68] Cross-sectional	China	Questionnaire (weekly MVPA via IPAQ-SF)	Questionnaire (SB via IPAQ-SF; time over the last7days spent on ST)	Questionnaire (average sleep time over past 7 days)	Depression, Anxiety, Stress (Chinese Version of DASS-21)	2476; Linear mixed models	M = 36	76.3% Female	Meeting the PA + ST*, ST + SL, or all 3 guidelines was associated with more favorable scores for depression compared to meeting none of the guidelines Compared to meeting none of the guidelines, meeting 1 or all 3* guidelines were associated with more favorable scores for depression Meeting the PA + ST or SL + ST* guidelines was associated with more favorable scores for anxiety compared to meeting none of the guidelines Compared to meeting none of the guidelines, meeting 2* or 3 guidelines was associated with more favorable scores for anxiety
Fung et al., (2023) [114] Cross-sectional and longitudinal	United States	Questionnaire (parent-reported days of physical activity per week with 60+ minutes)	Questionnaire (parent-report average daily time they spent engaging in recreational ST)	Questionnaire (parent-reported average daily hours of sleep)	Internalizing problems, total problems (CBCL)	10,574 (Baseline); 9273 (Follow-up); Linear mixed models	Baseline: M = 10; Follow-up: M = 12	48% Female (baseline); 47.7% Female (Follow-up)	Baseline: Meeting any combination of guidelines (all 3*) was associated with the more favorable scores for internalizing problems and total problems compared to meeting none of the guidelines Follow-up: Meeting SL + ST, ST + PA or all 3* guidelines were associated with more favorable scores for internalizing problems and total problems compared to meeting none of the guidelines T2-T1: Meeting ST + PA* guidelines was associated with more favorable scores for internalizing problems compared to meeting none of the guidelines, whereas meeting the SL + ST* guidelines were associated with more favorable scores for total problems
García- Hermosos (2022) [58] Cross-sectional and longitudinal	United States	Questionnaire (physical activity frequency)	Questionnaire (weekly hours of recreational ST)	Questionnaire (sleep duration)	Depression (self-reported diagnosis, symptoms and/or depression medication usage); Suicidal ideation	7069; Poisson regression	Baseline: M = 15; Follow-up: M = 37	56.8% Female	Baseline: Meeting all 3 guidelines was not associated with lower risk of depression compared to meeting none of the guidelines Meeting all 3* guidelines was associated with lower incidence rates of suicidal ideation compared to meeting none of the guidelines Follow-up: Meeting all 3* guidelines in adolescence and adulthood was associated with higher reduced risk of depression and suicidal ideation in adulthood
Gilchrist et al., (2021) [115] Cross-sectional	Canada	Questionnaire (MVPA using SHAPES)	Questionnaire (average daily time spent using devices)	Questionnaire (average hours of daily sleep)	Depressive symptoms (CESD-R-10); Anxiety symptoms (GAD-7); Flourishing (FS)	46,413; ISM	Grade 9–12	51.5% Female	< 8 h sleep: - + SL/-ST or MVPA: lower anxiety symptoms - + MVPA or SL/-ST: lower depressive symptoms - + SL/-MVPA: lower depressive symptoms - + MVPA or SL/-ST: greater flourishing scores - + MVPA/-SL: greater flourishing scores > = 8 h sleep: - + MVPA/-ST: lower anxiety symptoms - + ST/-SL: higher anxiety symptoms - + MVPA or SL/-ST: lower depressive symptoms - + MVPA or SL/-ST: greater flourishing scores - + MVPA/-SL: greater flourishing scores
Hajo et al., (2020) [116] Cross-sectional	Canada	Accelerometer (ActiGraph wGT3x—hip) LPA and MVPA	Accelerometer (ActiGraph wGT3x—hip) SB	Questionnaire (average hours of daily sleep)	Mood states (POMS)	342; Profiles with chi-squared tests	M = 43	94% Female	Engaging in healthy amounts of 3/3* movement behaviors, 2/3 behaviors and 0/3 behaviors were associated with more favorable scores than 1/3 behaviors
Hinkley et al., (2020) [117] Longitudinal	Australia	Accelerometer (ActiGraph GT1M – right hip)	Questionnaire (parent-reported average ST on weekdays and weekends)	Questionnaire (parent-reported average daily sleep)	Quality of Life (PQoL); Self-worth (SPPC); Total difficulties (SDQ)	1002 (T1), 567 (T2), 568 (T3); Linear and logistic regression	M = 4.6 (0.70) Range = 3–5	53% Female	Meeting all 3 guidelines was not associated with more favorable scores for quality of life, self-worth or total difficulties compared to meeting none of the guidelines
Hofman et al., (2022) [118] Cross-sectional	The Netherlands	Accelerometer (GENEActiv—non-dominant wrist) LPA and MVPA	Accelerometer (GENEActiv—non-dominant wrist) SB	Accelerometer (GENEActiv—non-dominant wrist) SL	Depressive symptoms (CESD); Anxiety (HADS)	1943; CoDA, including ISM	M = 71	52% Female	- + MVPA/-SL or SB: lower depressive symptoms - No effects of ISM on anxiety symptoms
Hou et al., (2023) [119] Cross-sectional	United States	Questionnaire (parent-reported days of physical activity per week with 60 or more minutes)	Questionnaire (parent-reported average daily hours of recreational screen time use)	Questionnaire (parent-reported hours of sleep most weeknights)	Depression and Anxiety (parent indicated whether the child received diagnosis from doctor); Resilience (parent indicated whether child stays calm and in control)	907; Multivariable Logistic Regression	M = 12.22 (2.78)	54.58% Female	Meeting SL + SB* guidelines had lower odds of depression and anxiety compared to meeting none of the guidelines Meeting the SL + PA* guidelines had higher odds of resilience compared to meeting none of the guidelines
Janssen et al., (2017) [69] Cross-sectional	Canada	Questionnaire (days of physical activity per week with 60 or more minutes; days per week of muscle strengthening activity)	Questionnaire (daily hours of ST)	Questionnaire (average weekday and weekend sleep duration)	Life satisfaction (Cantril ladder); Emotional problems (9 item scale)	20207–21821; Linear regression	Range = 10–17	52.8% Female	Emotional problems: Meeting all 3* guidelines < Meeting 2 guidelines < Meeting 1 guidelines < Meeting None Life satisfaction: Meeting all 3* guidelines > Meeting 2 guidelines > Meeting 1 guidelines > Meeting None Meeting any combination of guidelines was not associated with the more favorable scores for emotional problems or life satisfaction compared to meeting none of the guidelines
Kandola et al., (2021) [120] Longitudinal	United Kingdom	Accelerometer (Axivity AX3—wrist) LPA and MVPA	Accelerometer (Axivity AX3—wrist) SB	Questionnaire (average hours of daily sleep)	Depressive symptoms (PHQ-9); Anxiety symptoms (GAD-7)	60235; ISM	M = 55.9 (7.7)	56% Female	- + LPA, SL or MVPA/-SB: lower depressive symptoms - + LPA/-SB: higher anxiety symptoms - + MVPA or SL/-SB: lower anxiety symptoms
Kitano et al., (2020) [121] Cross-sectional	Japan	Accelerometer (Active Style Pro HJA750-C—hip) LPA and MVPA	Accelerometer (Active Style Pro HJA750-C—hip) SB	Questionnaire (average hours of daily sleep)	Psychological distress (K6)	1095; CoDA, including ISM	M = 50	68.6% Female	During workday: CoDA: The 24-h movement composition was significantly associated with psychological distress. Relative to other behaviors, SL was associated with lower psychological distress whereas SB and LPA were associated with greater psychological distress ISM:—+ SL/-SB or LPA: lower odds of psychological distress During non-workday: CoDA: The 24-h movement composition was not associated with psychological distress
Kuzik et al., (2020) [122] Cross-sectional	Canada	Accelerometer (ActiGraph wGT3x—hip) LPA and MVPA	Accelerometer (ActiGraph wGT3x—hip) SB	Accelerometer (ActiGraph wGT3x—hip) SL	Socio-emotional development (CSBQ)	95; CoDA, including ISM	Range = 3–5	30.5% Female	The 24-h movement composition was not associated with internalizing problems. Relative to other behaviors, MVPA was negatively associated with internalizing problems (when influential observations removed) ISM (30-min): - + MVPA/ -SB: lower internalizing problems scores (when influential observations removed) - + MVPA/-SL: lower internalizing problems scores (when influential observations removed)
Kuzik et al., (2022) [123] Cross-sectional	Canada	Accelerometer (ActiGraph WGT3X-BT—right hip)	Questionnaire (parent-reported, hours of screen time)	Accelerometer (ActiGraph WGT3X-BT—right hip)	Socio-emotional development (CSBQ)	95; Multiple regression	Range = 3–5	30.5% Female	Meeting any combination of guidelines was not associated with more favorable scores for internalizing problems compared to meeting none of the guidelines
Larisch et al., (2020) [124] Cross-sectional	Sweden	Accelerometer (ActiGraph wGT3x—right hip) LPA & MVPA	Accelerometer (ActiGraph wGT3x—right hip) SB	Accelerometer (ActiGraph wGT3x—wrist worn) SL	Anxiety and depressive symptoms (HADS); Well-being (WHO-Five well-being scale)	370; CoDA, including ISM	M = 41	68% Female	The 24-h movement composition was not associated with depression, anxiety or mental well-being. There were no effects of reallocating time across SL, SB, LPA, MVPA
Le et al., (2022) [125] Longitudinal	Australia	Accelerometer (ActiGraph wGT3x—wrist) LPA and MVPA	Accelerometer (ActiGraph wGT3x—wrist) SB	Accelerometer (ActiGraph wGT3x—wrist) SL	Daily affect (12-items from PANAS-E)	361: ISM	M = 22.62 (5.34)	72.6% Female	- + MVPA/-SL, SB or LPA: greater high arousal positive affect - No changes for high arousal negative affect, low arousal negative affect or low arousal positive affect
Lee et al., (2018) [126] Cross-sectional	South Korea	Questionnaire (days of physical activity per week with 60 or more minutes)	Questionnaire (average screen time duration per weekday and weekend)	Questionnaire (average daily sleep duration for weekdays and weekends)	Happiness (1- item measure)	50,987; Logistic regression	M = 15; Range = 12–17	49.0% Female	Meeting all 1, 2 or all 3* guidelines was associated with more favorable scores for happiness compared to meeting none of the guidelines (total guidelines) Meeting the PA + ST*, PA + SL, or all 3 guidelines were associated with more favorable scores for happiness compared to meeting none of the guidelines (specific guidelines)
Li et al., (2022) [70] Cross-sectional	7 countries: Brazil, Finland, Hong Kong, Mainland China, Singapore, South Korea, USA	Questionnaire (parent-reported days of physical activity per week with 60 or more minutes)	Questionnaire (parent-reported average ST)	Questionnaire (parent-reported average nightly sleep on weeknight)	Quality of life (1-item; parent reported satisfaction of life)	1165; Linear regression	M = 13.1 (2.2)	24.4% Female	Meeting an increasing number of guidelines was associated with more favorable scores for quality of life compared to meeting none of the guidelines Meeting any combination of guidelines was associated with similar benefits for quality of life compared to meeting all of the guidelines. Compared to meeting all three, meeting none was associated with lower quality of life
Liang et al., (2021) [64] Cross-sectional	China	Questionnaire (weekly MVPA via IPAQ-SF)	Questionnaire (SB via IPAQ-SF)	Questionnaire (Chinese version of the PSQI)	Depression (Chinese version of PHQ-9); Anxiety (SAS)	1846; MANCOVA	M = 21; Range = 18–26	64% Female	Meeting any combination of guidelines (all 3*) was associated with more favorable scores for depression compared to meeting none of the guidelines Meeting the SL + PA*, SL + SB, or all 3 guidelines was associated with more favorable scores for anxiety compared to meeting none of the guidelines
Liang et al., (2023) [127] Cross-sectional	China	Questionnaire (days of physical activity per week with 60 or more minutes)	Questionniare (time spent on screen-based activities)	Questionnaire (1-item from the Chinese version of the PSQI)	Well-being (Chinese version of the World Health Organization Five-Item Wellbeing Index); Resilience (Chinese version of the CD-RISC-10); depression (Chinese version of the PHQ-9); anxiety (Chinese version of the GAD-7)	67821; Linear regression	M = 13	48.1% Female	Specific combinations Meeting any combination of the guidelines (all 3*) was associated with more favorable scores for depression, well-being and resilience compared to meeting none of the guidelines Meeting any combination of the guidelines (ST + SL*) was associated with more favorable scores for anxiety compared to meeting none of the guidelines Total guidelines Meeting any number of guidelines (all 3*) was associated with more favorable scores for depression, anxiety, well-being and resilience compared to meeting none of the guidelines
Liu et al., (2022) [128] Cross-sectional	United States	Questionnaire (days of physical activity per week with 60 or more minutes)	Questionnaire (hours per day of recreational ST)	Questionnaire (hours of school-night sleep)	Suicidality (1 question for ideation, plan, attempt, and attempt with medical treatment in last 12 months)	73074; Logistic regression	Grades = 9–12	49.9% Female	Meeting all 3* guidelines was associated with lower odds of suicidal ideation and planning compared to meeting none of the guidelines for boys but not girls No differences in suicidal attempts were observed for different numbers of guideline adherence among boys and girls
López-Gil et al., (2022) [129] Cross-sectional	Spain	Questionnaire (weekly MVPA IPAQ-SF)	Questionnaire (parent-ST use on weekdays and weekends)	Questionnaire (parent-reported average daily sleep)	Total difficulties (SDQ)	3772; Logistic regression	M = 10; Range = 4–5 (preschool), 6–12 (children), 13–14 (adolescents)	49.4% Female	Meeting less than all 3 guidelines was associated with greater total difficulties compared to meeting all three guidelines among the total sample, males and females
Lu et al., (2021) [130] Cross-sectional	China	Questionnaire (days of physical activity per week with 60 or more minutes)	Questionnaire (HBSC average daily ST hours)	Questionnaire (HBSC average hours of sleep per night)	Depressive symptoms (Chinese version of PHQ-9); Anxiety (GAD-7)	5357; Logistic regression	M = 12	44.4% Female	Meeting ST + PA, SL + ST or all 3* were associated with lower depressive symptoms, whereas meeting SL + ST or all 3* were associated with lower anxiety symptoms compared to meeting none of the guidelines Meeting 1, 2 or 3* guidelines were associated with lower depressive and anxiety symptoms compared to meeting none of the guidelines
Luo et al., (2022) [131] Cross-sectional	China	Questionnaire (CNHS, time spent exercising in various sports were used to calculate meeting PA guideline)	Questionnaire (CHNS, time spent on screen, reading books, and other sedentary behaviors used to calculate meeting SB guideline)	Questionnaire (CHNS; self-reported amount of sleep each night)	Mental health (3-items assessing vitality, well-being and optimism)	4134; Logistic regression	M = 67	67.38% Female	Meeting 2 or all 3* guidelines were associated with more favorable mental health scores compared to meeting none of the guidelines Meeting the PA + SL* guidelines was associated with more favorable mental health scores compared to meeting none of the guidelines
Luo et al., (2023) [132] Cross-sectional	China	Questionnaire (frequency and duration of PA)	Questionnaire (average daily time spent using screen-based devices)	Questionnaire (daily sleep duration)	Depression (PHQ-9); Anxiety (GAD-7)	9420; Logistic regression	M = 14.53 (0.69)	45.2% Female	Meeting the PA + SL, ST + SL, or all 3* had lower odds of depression and anxiety compared to not meeting the guidelines
McNeill et al., (2020) [74] Cross-sectional and longitudinal	Australia	Accelerometers (ActiGraph GT3X +—right hip) MVPA	Questionnaire (parent-reported recreational ST on weekdays and weekends)	Questionnaire (parent-reported average daily sleep duration)	Total difficulties (Educator-reported version of the SDQ)	247; Linear Regression	M = 4; Range = 3–5	40% Female	Meeting any number or specific combination of guidelines was not associated with more favorable scores for total difficulties compared to meeting none of the guidelines
Meyer et al., (2020) [133] Cross-sectional and longitudinal	United States	Accelerometer (SenseWear—armband) LPA and MVPA	Accelerometer (SenseWear—armband) SB	Accelerometer (SenseWear—armband) SL	Mood (POMS)	423; ISM	M = 28; Range: 21–35	50% Female	Baseline: -No effects of replacing Total SB with SL, LPA, or MVPA 12-months: - + MVPA or LPA/-SB: more favorable mood scores
Ohta et al., (2023) [134] Cross-sectional	Japan	Questionnaire (GPAQ, Japanese version)	Questionnaire (GPAQ, Japanese version)	Questionnaire (typical hours and minutes of nightly sleep)	Depressive symptoms (CES-D)	640; Logistic regression	M = 64.1 (14.0)	58.3% Female	Meeting the SB + PA or all 3* guidelines were associated with lower odds of depression compared to not meeting the guidelines Meeting 2 or 3* guidelines was associated with lower odds of depression compared to not meeting the guidelines
Peralta et al., (2022) [71] Cross-sectional	Switzerland	Questionnaire (average hours per week spent in MVPA)	Questionnaire (hours spent on electronic media on weekday and weekend)	Questionnaire (average hours spent asleep on weekday and weekend)	Life Satisfaction (Cantril ladder)	2534; Linear regression	Range = 5—16	51.5% Female	Meeting 2 or all 3* guidelines was associated with greater life satisfaction scores compared to meeting none of the guidelines Meeting the PA + ST*, ST + SL, or all 3 guidelines was associated with greater life satisfaction scores compared to meeting one guideline
Perez et al., (2022) [135] Cross-sectional	United States	Questionnaire (NHANES two items to assess MVPA in last 30 days)	Questionnaire (average hours of screen use per day in last 30 days)	Questionnaire (average hours of sleep in 24 h period)	PTSD (Primary Care PTSD; PC-PTSD-5); Suicide Ideation (item from NSDUH); Psychological Distress (K6)	17166; Logistic regression and multivariate models	Range = 18–45 +	16.7% females (weighted)	Meeting all 3* or some of the guidelines were associated with more favorable scores for psychological distress and PTSD compared to meeting none of the guidelines among men Meeting some of the guidelines was associated with more favorable scores for psychological distress among women Meeting all three* or some of the guidelines was associated with more favorable scores for suicidal ideation compared to meeting none of the guidelines for men and women
Porter et al., (2023) [136] Cross-sectional	Canada	Questionnaire (IPAQ)	Questionnaire (average daily hours of recreational ST via International Sedentary Assessment Tool); Questionnaire (average daily sitting time—SB)	Questionnaire (average hours of nightly SL)	Psychological distress (Kessler-10); Mental wellbeing (WEMWS)	17874; Linear regression (Propensity score weighted)	M = 21.6 (2.94)	65.2% Female	Meeting all 3* guidelines concurrently was associated with more favorable scores for psychological distress and mental well-being for those with and without chronic health conditions and disabilities
Sampasa-Kanyinga et al., (2020) [137] Cross-sectional	Canada	Questionnaire (days of physical activity per week with 60 or more minutes)	Questionnaire (average daily time spent using devices in last 7 days)	Questionnaire (average nightly sleep on school night)	Suicidal Ideation (1 item); Suicide Attempts (1 item)	10183; Logistic regression	M = 15	49% Female	Meeting any combination of the guidelines was not associated with differences in suicidal ideation and attempts compared to meeting none of the guidelines among boys ages 11–14 years Meeting all 3* guidelines was associated with more favorable scores for suicidal ideation and attempts compared to meeting none of the guidelines for boys ages 15–20 years Meeting the PA + ST* guidelines was associated with more favorable scores for suicidal ideation compared to meeting none of the guidelines among girls ages 11–14 years, but no differences were observed for suicidal attempts Meeting the PA + SL* guidelines was associated with more favorable scores for suicidal ideation compared to meeting none of the guidelines among girls ages 11–14 years, but no differences were observed for suicidal attempts
Sampasa-Kanyinga, Colman, Dumuid, et al., (2021) [138] Longitudinal	Canada	Questionnaire (average daily MVPA)	Questionnaire (average daily time they spent using devices during their free time)	Questionnaire (average hours of daily sleep)	Depressive symptoms (CESD-R-10)	14620 (2,836 younger boys, 2264 older boys, 5060 younger girls, 4460 older girls); CoDA, including ISM	M = 15	46% Female	Younger boys: CoDA: Relative to other behaviors, a favorable association was observed for SL with depressive symptoms, whereas an adverse association was observed for ST ISM: - + SL/-MVPA or ST: lower depressive symptoms Younger girls and older boys: - CoDA: Relative to other behaviors, a favorable association was observed for SL with depressive symptoms, whereas an adverse association was observed for ST - + SL/-MVPA or ST: lower depressive symptoms - + MVPA/-ST: lower depressive symptoms Older girls: - Relative to other behaviors, favorable association was observed for SL and MVPA with depressive symptoms, whereas an adverse association was observed for ST - + SL or MVPA/-ST: lower depressive symptoms - + SL/-MVPA: higher depressive symptoms
Sampasa-Kanyinga, Chaput, et al., (2021) [139] Cross-sectional	Canada	Questionnaire (days of physical activity per week with 60 or more minutes)	Questionnaire (average daily time they spent using devices during their free time)	Questionnaire (average nightly sleep on weekdays and weekend days)	Depression and Anxiety (K6)	6364; Structural equation modeling	M = 15.1 (1.8)	48.3% Female (weighted)	Meeting the ST + SL*, PA + SL, or all 3 guidelines were associated with more favorable scores for anxiety symptoms compared to meeting none of the guidelines Meeting any combination of guidelines (ST + SL*) was associated with more favorable scores for depression symptoms compared to meeting none of the guidelines
Sampasa-Kanyinga, Colman, Goldfield, et al., (2021) [140] Cross-sectional	Canada	Questionnaire (YRBSS; days of physical activity per week with 60 or more minutes)	Questionnaire (YRBS; average hours of ST per day)	Questionnaire (parent-reported number of hours of sleep child gets on most nights)	Internalizing problems (parent-reported CBCL)	11875; Negative binomial regression	M = 10; Range = 9–11	47.9% Female	Meeting any combination of guidelines (all 3*) was associated with more favorable scores for internalizing problems compared to meeting none of the guidelines
Sampasa-Kanyinga et al., (2022a) [72] Cross-sectional	Canada	Questionnaire (YRBSS; days of physical activity per week with 60 or more minutes; weekly frequency of muscle strengthening activity)	Questionnaire (YRBS; average hours of ST per day)	Questionnaire (hours of school-night sleep)	Self-Esteem (RSE)	6932; Logistic regression	M = 15; Range = 11–20	56.8% Female	Meeting any combination of guidelines (all 3*) was associated with more favorable scores for self-esteem compared to meeting none of the guidelines Meeting 1, 2 or all 3* guidelines were associated with more favorable scores for self-esteem compared to meeting none of the guidelines
Sampasa-Kanyinga et al., (2022b) [141] Cross-sectional	Canada	Questionnaire (days of physical activity per week with 60 or more minutes)	Questionnaire (average hours of ST per day)	Questionnaire (hours of school-night sleep)	Mental health (1 item)	5739 (2017), 6960 (2019); Logistic regression	M = 15.2 (1.8)	51% Female (2017); 50.6% Female (2019)	Meeting 1, 2 or all 3* guidelines were associated with more favorable scores for mental health compared to meeting none of the guidelines among the 2017 and 2019 samples Meeting any combination of guidelines was associated with more favorable scores for mental health compared to meeting none of the guidelines among the 2017 (PA + SL*) and 2019 (all 3*) samples
St. Laurent, et al., (2023) [142] Cross-sectional	United States	Accelerometer (Actiwatch Spectrum—non-dominant wrist) LPA and MVPA	Accelerometer (Actiwatch Spectrum—non-dominant wrist) SB	Accelerometer (Actiwatch Spectrum – non-dominant wrist) SL	Internalizing behaviors (CBCL)	388; CoDA, compositional linear regression	M = 51.5 months (9.46); R = 33 – 70 months	44.4% Female	No association was observed for the 24-h composition with internalizing behaviors
Sun et al., (2023) [143] Cross-sectional	China	Questionnaire (days of physical activity per week with 60 or more minutes)	Questionnaire (HBSC average daily ST hours)	Questionnaire (Chinese version of the PSQI)	Subjective well-being (WHO-5)	1098; Linear regression	M = 11.6 (0.8)	48.5% Female	Meeting 1, 2 or all 3* guidelines were associated with more favorable scores for subjective well-being compared to meeting none Meeting the PA + SL*, PA + SB or all 3 guidelines were associated with more favorable scores for subjective well-being compared to meeting none
Taylor 2021 [75] Longitudinal	New Zealand	Accelerometer (ActiCal—waist) LMVPA	Accelerometer (ActiCal—waist) SB; Questionnaire (parent-reported average daily time spent using devices) ST	Accelerometer (ActiCal—waist) SL	Anxiety, Depression & Resilience (BASC-2: 2–5 year old scale)	528; Linear regression	Range = 1- 5	48.5% Female	Meeting all 3* guidelines was associated with more favorable scores for depression at 5 years of age compared to meeting none of the guidelines, but no differences were observed at age 1 and 3 years Meeting all 3* guidelines at 1 year of age was associated with more favorable scores for anxiety at 5 years of age compared to meeting none of the guidelines, but no differences were observed when meeting guidelines at age 3 and 5 years Meeting all 3* guidelines was not associated with differences in resilience at 1, 3 and 5 years of age compared to meeting none of the guidelines
Taylor et al., (2023) [144] Cross-sectional and longitudinal	New Zealand	Accelerometer (Actical—waist) LMVPA	Accelerometer (Actical—waist) SB	Accelerometer (Actical—waist)	Anxiety, Depression & Resilience (BASC-2)	392; CoDA; Linear regression	Range = 2–5	49.7% Female	Cross-sectional: Relative to other behaviors, a favorable association was observed for MVPA with anxiety and resilience Longitudinal: No significant associations were observed for mental health at 2 and 3.5 years of age compared to 5 years
Vanderlinden et al., (2023) [145] Cross-sectional	Belgium	Accelerometer (Actigraph wGT3X-BT—non-dominant wrist) LMVPA	Accelerometer (Actigraph wGT3X-BT—non-dominant wrist) SB	Accelerometer (Actigraph wGT3X-BT—non-dominant wrist) SL	Mental wellbeing (WEMWBS)	410; CoDA, including ISM	M = 71.3 (6.3)	71% Female	CoDA: No behaviors were associated with well-being in the full-adjusted model ISM: No significant associations
Wang et al., (2022) [73] Cross-sectional	United States	Questionnaire (parent-reported days of physical activity per week with 60 or more minutes)	Questionnaire (parent-reported average screen time per day in last week)	Questionnaire (parent-reported average hours of sleep on a weeknight in last week)	Flourishing (3 questions; parent-reported)	634; Logistic regression	M = 14; Range = 10–17	29.3% Female	Meeting an increasing number of the guidelines was associated with more favorable flourishing scores compared to meeting none of the guidelines (total guidelines) Meeting all 3* guidelines was associated with more favorable flourishing scores (specific guidelines)
Zhang et al., (2022) [146] Cross-sectional	China	Questionnaire (LPA & MVPA via the PPAQ-C)	Questionnaire (SB via the PPAQ-C)	Questionnaire (typical hours of nightly sleep)	Anxiety (GAD-7)	873; ISM	Range = < 30—> 35	100% Female	Full sample: - + MVPA, SL or SB/-LPA: lower anxiety scores - + SL/-MVPA or SB: lower anxiety scores < 7 h nightly sleep: - No effects for reallocating time across behaviors > = 7 h nightly sleep: - No effects for reallocating time across behaviors
Zhang et al., (2023) [147] Cross-sectional and longitudinal	China	Questionnaire (daily MVPA via IPAQ-SF)	Questionnaire (average hours of recreational ST)	Questionnaire (typical hours of nightly sleep)	Depression (PHQ-9); Anxiety (GAD-7)	906; Linear regression	M = 14.3 (0.9)	49.0% Female	Cross-sectional: Meeting 1, 2 or all 3* guidelines were associated with lower depression and anxiety symptoms compared to meeting none of the guidelines Meeting PA + ST, ST + SL, or all 3* guidelines were associated with lower depression and anxiety symptoms compared to meeting none of the guidelines Longitudinal: Meeting 1, 2, or all 3* guidelines were associated with lower depression and anxiety symptoms compared to meeting none of the guidelines 6 months later Meeting ST + SL or all 3* guidelines were associated with lower depression and anxiety symptoms compared to meeting none of the guidelines 6 months later
Zhu et al., (2019) [61] Cross-sectional	United States	Questionnaire (parent-reported days of physical activity per week with 60 or more minutes)	Questionnaire (parent-reported average screen time per day in last week)	Questionnaire (parent-reported average hours of nightly sleep)	Anxiety and depression (parent indicated whether child received diagnosis from doctor)	20,708; Logistic regression	Range = 5–17	49.0% Female (6–11 years); 48.9% Female (12 -17 years)	Meeting any combination of guidelines (all 3*) was associated with reduced risk of depression compared to meeting none of the guidelines among 12–17 year olds, whereas meeting the PA + SL* and ST + SL guidelines was favorable for 6–11 year olds Meeting all 3* guidelines was associated with reduced risk of anxiety compared to meeting none of the guideline among 12–17 year olds, whereas no differences were observed for guideline adherence among 6–11 year olds
Zhu et al., (2023) [148] Cross-sectional	China	Accelerometer (Active Style Pro HJA-750C)	Questionnaire (parent-reported hours per day of recreational ST)	Questionnaire (parent-reported average nightly sleep)	Internalising problems (SDQ—Chinese version)	200; Logistic regression	M = 57.5 months (10.0 months)	49% Female	Weekend: Meeting no guidelines*, PA + SL, or PA + ST had the higher odds of internalizing problems compared to meeting all 3 guidelines Weekday: Meeting any specific combination of guidelines was not associated lower odds of internalizing problems compared to meeting all 3 guidelines

BASC-2: Behavioral Assessment System for Children; BMDSLSS: Brief Multi-Dimensional Students' Life Satisfaction Scale; CBCL: Child Behavior Checklist; CCD: Chronic health Conditions and Disabilities; CD-RISC-10: Connor-Davidson Resilience Scale 10-item; CES-D: Center for Epidemiological Studies-Depression; CESD-R: Centre of Epidemiologic Studies Depression Scale–Revised; CHNS: Chinese Health and Nutrition Survey; CoDA: compositional data analysis; CSBQ: Child Self-Regulation and Behaviour Questionnaire; DASS-21: Depression Anxiety Stress Scales-21 items; DERS: Difficulties in Emotion Regulation Scale-Short Form; FS: Flourishing Scale; GAD-7: Generalized Anxiety Disorder Scale; GHQ-12: General Health Questionnaire; GPAQ: Global Physical Activity Questionnaire; HADS: Hospital Anxiety and Depression Scale; HBSC: Health Behavior in School-aged Children; IPAQ-SF: International Physical Activity Questionnaire-Short Form; ISM: isotemporal substitution analysis; K6/K10: Kessler Psychological Distress Scale; MFQ-Short: Short Mood and Feelings Questionnaire; NSDUH: National Survey on Drug Use and Health; PANAS-E: Positive and Negative Affect Schedule-Expanded; PC-PTSD-5: Primary Care PTSD Screen for DSM-5; POMS: Profile of Mood States; PPAQ-C: Chinese version of the Pregnancy Physical Activity Questionnaire; PSQI: Pittsburgh Sleep Quality Inventory; RSE: Rosenberg Self-Esteem Scale; SAS: Zung's Self-rating Anxiety Scale; SDQ: Strengths and Difficulties Questionnaire; SHAPES: School Health Action, Planning and Evaluation System questionnaire; SPPC: Harter’s Self-Perception Profile for Children; WEMWBS: Warwick-Edinburgh Mental Wellbeing Scale

^*^The most favorable association

### Measurement of movement behaviors

Physical activity was assessed via accelerometers in 31 studies (*n* = 16 for children and youth; *n* = 15 for adults) and using self- or proxy-reported questionnaires in 42 studies (n = 31 for children and youth; *n* = 11 for adults). Sedentary behavior, including recreational screen time, was assessed via accelerometers in 21 studies (*n* = 7 for children and youth; *n* = 14 for adults), self- or proxy-reported questionnaires in 50 studies (*n* = 38 for children and youth;* n* = 12 for adults), and a combination of accelerometers and self-reported questionnaires in two studies that were both conducted with samples of children and youth. Finally, sleep was assessed via accelerometers in 20 studies (*n* = 11 for children and youth; n = 9 for adults) and using self- or proxy-reported questionnaires in 53 studies (*n* = 36 for children and youth; *n* = 17 for adults).

### Indicators of mental ill-being and well-being

Most studies (44 of 73; 60%) assessed multiple indicators of mental health. Indicators of mental well-being (i.e., positive mental health) included: flourishing (*n* = 5 for children and youth), quality of life (*n* = 3 for children and youth), self-esteem (*n* = 5 for children and youth), resilience (*n* = 6 for children and youth), life satisfaction (*n* = 3 for children and youth), general mental health (*n* = 2 for children and youth; *n* = 4 for adults), happiness (*n* = 1 for children and youth; *n* = 1 for adults), well-being (*n* = 1 for children and youth; *n* = 4 for adults), daily affect (*n* = 2 for adults) and mood states (*n* = 1 for adults). Indicators of mental ill-being (i.e., negative mental health) included: depression or depressive symptoms (*n* = 18 for children and youth; *n* = 13 for adults), anxiety or anxiety symptoms (*n* = 13 for children and youth; *n* = 10 for adults), psychological distress (*n* = 2 for children and youth; *n* = 4 for adults), loneliness (*n* = 1 for children and youth; *n* = 1 for adults), prolonged sadness (*n* = 1 for children and youth), suicidal ideation and planning (*n* = 2 for children and youth; *n* = 3 for adults), suicidal attempts (*n* = 2 for children and youth), total difficulty problems (*n* = 11 for children and youth), internalizing problems (*n* = 11 for children and youth), emotional dysregulation (*n* = 1 for children and youth), and post-traumatic stress disorder (*n* = 1 for adults). More associations were reported for indicators of mental ill-being (*n* = 127 for children and youth; *n* = 53 for adults) than well-being (*n* = 54 for children and youth; *n* = 26 for adults).

Some studies stratified their results by different factors including sex/gender (*n* = 5 studies), age groups including school grade (*n* = 6 studies), sleep guideline adherence (*n* = 2 studies), weight status (*n* = 1), workday vs. non workday (*n* = 1), weekday vs. weekend (*n* = 1), and chronic health conditions and disabilities (CCD; *n* = 1). As a result, total associations are out of the number of individual samples (i.e., a study that stratified their sample by sex/gender would be counted as two associations: one for boys and one for girls), rather than number of studies.

### Associations between combinations of 24-h movement behaviors and mental health

A summary of all associations by age group (children and youth; adults), analytic approach, indicator of mental health (ill-being; well-being), and design (cross-sectional; longitudinal) is presented in Additional file 3.

#### Cross-sectional studies of children and youth

*Total guideline adherence* Among cross-sectional studies, 24/38 (63%) of the associations showed that meeting all three guidelines concurrently was correlated with favorable scores for indicators of mental health, whereas 22/38 (58%) and 23/38 (60%) associations demonstrated favorable effects for meeting one guidelines or two guidelines, respectively. When decomposed into indicators of well-being and ill-being, findings revealed 10/14 (71%), 11/14 (79%), and 11/14 (79%) associations were favorable effects when meeting one, two, or three guidelines, respectively, for indicators of mental well-being. For indicators of mental ill-being, 13/24 (54%) associations demonstrated favorable effects for meeting all three guidelines, 12/24 (50%) associations for meeting two guidelines, and 12/24 (50%) for meeting only one guideline.

*Specific combinations of guideline adherence* A total of 33/58 (57%) associations showed favorable effects for meeting all three guidelines concurrently. In contrast, 33/58 (57%), and 25/58 (43%), 25/58 (43%), associations demonstrated favorable effects for adherence to the screen time and sleep, physical activity and sleep, and physical activity and screen time guidelines, respectively. Specific to indicators of mental well-being, findings revealed 10/13 (77%) associations were favorable effects for adherence to all three guidelines, 8/13 (62%) for physical activity and sleep guideline adherence, 8/13 (62%) for physical activity and screen time guideline adherence, and 6/13 (46%) for screen time and sleep guideline adherence. For indicators of mental ill-being, findings revealed 23/45 (51%) associations were favorable effects for adherence to all three guidelines, 27/45 (60%) for meeting the screen time and sleep guidelines, 17/45 (38%) associations for meeting the physical activity and sleep guidelines and 17/45 (38%) associations for meeting the physical activity and screen time guidelines.

*Compositional data analysis* A significant relationship between the 24-h movement composition and indicators of mental health was observed in 11/20 (55%) associations. Among the studies that reported the effects of each behavior (relative to others), significant adverse associations were observed for light physical activity (2/16; 13%) and sedentary behavior (9/16; 56%), whereas significant beneficial associations were observed for sleep (6/16; 38%), MVPA (4/16; 25%) and light physical activity (1/16; 6%). Examining indicators of mental well-being and ill-being independently revealed a significant relationship for the 24-h movement composition with indicators of mental well-being in 4/7 (57%) associations and 7/13 (54%) associations for indicators of mental ill-being. Among the studies that reported the effects of each behavior (relative to others), significant favorable associations were observed for MVPA (1/3; 33%) for indicators of mental well-being and for sleep (6/13; 46%), MVPA (3/13; 23%), and light physical activity (1/13; 7%) for indicators of mental ill-being. Whereas significant adverse effects were observed for light physical activity (2/13; 15%) and sedentary behavior (9/16; 70%) for indicators of mental ill-being.

*Goldilocks method* The optimal 24-h movement composition for mental health was roughly 10 to 11 h of sleep; 6.5 to 8 h of sedentary time, 2 to 7 h of light physical activity, and 43 min to 2 h of MVPA in 5/5 (100%) associations. These results were consistent across the two associations for indicators of mental well-being and three associations for mental ill-being.

*Rest-activity rhythmicity* A significant negative relationship (more stability) was observed between inter-daily stability and indicators of mental ill-being in 1/2 (50%) associations, whereas no associations (0/2; 0%) were significant for intra-day variability. Rest-activity rhythmicity has not been examined in relation to mental well-being, to date.

*Isotemporal substitution* Replacing sedentary behavior with MVPA (14/17 associations; 82%) or sleep (13/17 associations; 76%) were most consistently found to be correlated with more favorable mental health scores. Reallocating sleep to MVPA was correlated with more favorable mental health scores in 4/17 (26%) associations, whereas in contrast, reallocating MVPA to sleep was correlated with more favorable mental health scores in 5/17 (29%) associations. When decomposed into indicators of well-being and ill-being, results revealed similar patterns. For mental well-being, replacing sedentary behavior with MVPA (7/8 associations; 88%) or sleep (6/8 associations; 75%) had favorable effects. For mental ill-being, replacing sedentary behavior with MVPA (8/9 associations; 89%) or sleep (7/9 associations; 78%) was correlated with favorable scores. Reallocating time from sleep to MVPA was beneficial in 3/8 associations (38%) for mental well-being compared to 1/9 associations (11%) for mental ill-being. Replacing MVPA with sleep was only beneficial for indicators of mental ill-being (5/9 associations; 56%).

*Latent profile or cluster-based analyses* The healthiest combination of movement behaviors (adequate sleep, high MVPA, low sedentary time) was correlated with the most favorable mental health scores across 5/5 associations (100%), followed by mixed behavioral profiles (e.g., healthy amounts of sleep and MVPA, but not screen time), and lastly, consistently unhealthy profiles (e.g., least healthy amounts of all three movement behaviors). These findings were consistent for 3/3 (100%) associations investigating indicators of mental well-being and 2/2 (100%) associations investigating mental ill-being.

#### Longitudinal studies of children and youth

*Total guideline adherence* For total guideline adherence, meeting one, two or three guidelines concurrently was correlated with favorable scores for indicators of mental health in 3/12 (25%) associations. None of the associations were significant for indicators of mental well-being (0/5; 0%), whereas 3/7 (43%) associations showed favorable effects for indicators of mental ill-being.

*Specific combinations of guideline adherence* Favorable mental health scores were found in 2/4 (50%) associations for meeting all three guidelines, 1/4 (25%) associations for meeting the screen time and physical activity guidelines and 3/4 (75%) associations for screen time and sleep guideline adherence. All associations investigated indicators of mental ill-being.

*Compositional data analysis* No associations (0/5; 0%) demonstrated a significant relationship for the 24-h movement composition with indicators of mental well-being (0/2 associations; 0%) or mental ill-being (0/3 associations; 0%).

*Isotemporal substitution* Akin to the cross-sectional studies among children and youth, replacing sedentary behavior with MVPA (8/8 associations; 100%) or sleep (7/8 associations; 89%) were most consistently found to be correlated with more favorable mental health scores. Comparatively, 5/8 (63%) associations found that replacing MVPA with sleep was correlated with better scores for mental health, whereas only 2/8 (26%) associations showed replacing sleep with MVPA was correlated with more favorable mental health scores. All associations investigated indicators of mental ill-being.

*Latent profile or cluster-based analyses* The healthiest combination of movement behaviors (adequate sleep, high MVPA, low sedentary time) was associated with the most favorable mental ill-being scores in 1/1 (100%) associations, whereas no differences were observed across the other behavioral combination profiles. Associations with indicators of mental well-being have not been examined.

#### Cross-sectional studies of adults

*Total guideline adherence* For total guideline adherence, 13/21 (62%) associations showed that meeting all three guidelines concurrently was associated with favorable effects for indicators of mental health, whereas 8/21 (38%) and 3/21 (14%) associations showed meeting two guidelines, or one guideline were associated with favorable effects, respectively. For indicators of well-being, findings revealed 1/7 (14%), 1/7 (14%), and 3/7 (43%) associations were favorable effects for adherence to one, two, and all three guidelines, respectively. For indicators of mental ill-being, a total of 2/14 (14%), 7/14 (50%), and 10/14 (71%) associations were favorable effects for adhering to one, two, and all three guidelines, respectively.

*Specific combinations of guideline adherence* A total of 5/8 (63%) associations demonstrated favorable effects for meeting all three guidelines concurrently, whereas 5/8 (63%), 5/8 (63%), and 4/8 (50%) associations were favorable effects for adherence to the physical activity and sleep guidelines, sedentary behavior and sleep, and physical activity and sedentary behavior guidelines, respectively. For indicators of mental well-being, results indicated a favorable effect for adhering to the physical activity and sleep guidelines in 1/1 (100%) associations. For mental ill-being, findings revealed 4/7 (57%), 4/7 (57%), 5/7 (71%), and 5/7 (71%) associations were favorable effects for adherence to the physical activity and sleep guidelines, physical activity and sedentary behavior guidelines, sleep and sedentary behavior guidelines, and all three guidelines, respectively.

*Compositional data analysis* A significant relationship between the 24-h movement composition and mental health was observed in 4/13 (31%) associations. Among the studies that reported the effects of each behavior (relative to others), significant favorable associations were observed for MVPA (4/10; 40%), sleep, (1/10; 10%) and light physical activity (1/10; 10%), whereas significant adverse associations were observed for sedentary behavior (2/10; 20%) and light physical activity (1/10; 10%). Among these results, findings revealed a significant relationship between the 24-h movement composition and indicators of mental well-being in 1/4 (25%) associations and 3/9 (33%) associations for indicators of mental ill-being. For studies that reported the effects of each behavior (relative to others), significant favorable effects were observed for MVPA (3/5; 60%) and light physical activity (1/5; 20%) for indicators of mental well-being and favorable effects were observed for MVPA (1/5; 20%) and sleep (1/5; 20%) for indicators of mental ill-being. Whereas adverse associations were observed for sedentary behavior (2/5; 40%) and light physical activity (1/5; 20%) for indicators of mental ill-being.

*Isotemporal substitution* Replacing sedentary behavior with MVPA was associated with more favorable mental health scores in 8/17 (47%) associations. Reallocating time from sedentary behavior (6/17 associations; 35%) or MVPA (1/17 associations; 6%) to sleep was found to have beneficial effects for mental health. Replacing sleep with MVPA was also found to be correlated with more favorable benefits for mental health in 5/17 (29%) associations. Finally, reallocating time from sleep (4/17; 24%) or sedentary behavior (5/14; 29%) to LPA was found to have beneficial effects for mental health. Examining indicators of mental well-being and ill-being independently revealed similar pattens. For mental well-being, replacing sedentary behavior with MVPA (2/5 associations; 40%) or sleep (2/5 associations; 40%) was associated with more favorable effects. Reallocating time from sleep (2/5 associations; 40%) or sedentary behavior (2/5 associations; 40%) to LPA was also found to be correlated with favorable benefits for mental well-being. For indicators of mental ill-being, replacing sedentary behavior with MVPA (6/12 associations; 50%) or sleep (4/12 associations; 33%) was associated with more favorable effects. Reallocating time from sleep (2/12 associations; 17%) or sedentary time (3/12 associations; 25%) to LPA was also correlated with more favorable scores for mental ill-being.

*Latent profile or cluster-based analyses* A total of 2/3 (67%; 2/2 for indicators of mental well-being) associations revealed the healthiest combinations of movement behaviors (adequate sleep, high MVPA, low sedentary time) was associated with the most favorable scores for mental health, followed by mixed behavioral profiles, and lastly, consistently unhealthy profiles. For indicators of mental ill-being, a total of 1/3 (33%) associations showed engaging in healthy amounts of all three behaviors, two out of three behaviors or no behaviors were associated with more favorable scores than engaging in healthy amounts of only one behavior.

#### Longitudinal studies of adults

*Total guideline adherence* For total guideline adherence, 2/2 (100%) associations (both measures of mental ill-being) showed that meeting all three guidelines concurrently was associated with favorable effects for mental health.

*Compositional data analysis* No associations (0/4; 0%) found a significant relationship between the 24-h movement composition and indicators of mental well-being (0/2 associations; 0%) or mental ill-being (0/2 associations; 0%).

*Isotemporal substitution* Replacing sedentary behavior with MVPA was correlated with more favorable mental health scores in 5/11 (45%) associations, whereas replacing sedentary behavior with sleep was only correlated with beneficial effects for mental health scores in 2/11 (18%) associations. Finally, 2/11 (18%) associations revealed that reallocating time from sleep to MVPA was associated with beneficial effects for mental health. For indicators of well-being, beneficial effects were observed in 3/5 (60%) associations when replacing sedentary time for MVPA. Reallocating time from sleep to MVPA was associated with beneficial effects for mental well-being in 2/5 (40%) associations. For mental ill-being, replacing sedentary time with MVPA (2/6 associations; 33%) or sleep (2/6 associations; 33%) was correlated with more favorable scores.

### Methodological quality and risk of bias assessment

The study quality and risk of bias results are presented in Table 2. The majority of studies (*n* = 66; 90%) were considered to be of fair quality, with only 7 (10%) considered to be of good quality. No studies were considered of poor quality.

**Table 2:** Risk of bias

	Baillot et al. (2022)	Bang et al. (2020)	Blodgett et al. (2023)	Brown et al. (2021) MHAPA	Brown et al., (2021) PM	Brown et al., (2021) FIBN	Brown et al. (2021) JoPA	Brown et al., (2021) E&B	Brown et al. (2022) JoAD	Brown et al. (2022) MHaPH	Bu et al., (2021)
1. Was the research question or objective in this paper clearly stated?	Yes	Yes	Yes	Yes	Yes	Yes	Yes	Yes	Yes	Yes	Yes
2. Was the study population clearly specified and defined?	Yes	Yes	Yes	No	Yes	No	Yes	Yes	Yes	Yes	Yes
3. Was the participation rate of eligible persons at least 50%?	No	No	Yes	No	No	No	Yes	Yes	No	No	Yes
4. Were all the subjects selected or recruited from the same or similar populations (including the same time period)? Were inclusion and exclusion criteria for being in the study prespecified and applied uniformly to all participants?	Yes	Yes	Yes	Yes	Yes	Yes	Yes	Yes	Yes	Yes	Yes
5. Was a sample size justification, power description, or variance and effect estimates provided?	No	No	No	No	No	No	No	No	No	No	No
6. For the analyses in this paper, were the exposure(s) of interest measured prior to the outcome(s) being measured?	No	No	No	No	Yes	No	No	No	No	No	No
7. Was the timeframe sufficient so that one could reasonably expect to see an association between exposure and outcome if it existed?	No	No	No	No	Yes	No	No	No	No	No	No
8. For exposures that can vary in amount or level, did the study examine different levels of the exposure as related to the outcome (e.g., categories of exposure, or exposure measured as continuous variable)?	Yes	Yes	Yes	Yes	Yes	Yes	Yes	No	Yes	Yes	No
9. Were the exposure measures (independent variables) clearly defined, valid, reliable, and implemented consistently across all study participants?	Yes	Yes	Yes	Yes	Yes	Yes	Yes	Yes	Yes	Yes	Yes
10. Was the exposure(s) assessed more than once over time?	No	No	No	No	No	No	No	No	No	No	No
11. Were the outcome measures (dependent variables) clearly defined, valid, reliable, and implemented consistently across all study participants?	Yes	Yes	Yes	Yes	Yes	Yes	Yes	Yes	Yes	Yes	Yes
12. Were the outcome assessors blinded to the exposure status of participants?	Other	Other	Other	Other	Other	Other	Other	Other	Other	Other	Other
13. Was loss to follow-up after baseline 20% or less?	Other	Other	Other	Other	No	Other	Other	Other	Other	Other	Other
14. Were key potential confounding variables measured and adjusted statistically for their impact on the relationship between exposure(s) and outcome(s)?	Yes	Yes	Yes	Yes	Yes	Yes	Yes	Yes	Yes	Yes	Yes
Totals	7	7	7	6	9	6	8	7	7	7	7

	Burns et al., (2020)	Cabanas-Sánchez et al., (2021)	Cao et al., (2020)	Carson et al., (2019)	Chao et al. (2022)	Chong et al., (2021)	Christia n et al., (2022)	Colley et al., (2018)	Curtis et al., (2020)	Curtis et al., (2023)	del Pozo Cruz et al.,	Dumid et al., (2022)	Duncan et al. (2022)	Fairclough et al., (2021)	Fairclough et al., (2023)	Faria et al. (2022)	Feng et al., (2022)
1. Was the research question or objective in this paper clearly stated?	Yes	Yes	Yes	Yes	Yes	Yes	Yes	Yes	Yes	Yes	Yes	Yes	Yes	Yes	Yes	Yes	Yes
2. Was the study population clearly specified and defined?	Yes	Yes	Yes	Yes	No	Yes	Yes	Yes	Yes	Yes	Yes	Yes	Yes	Yes	Yes	Yes	Yes
3. Was the participation rate of eligible persons at least 50%?	Yes	No	Yes	No	Yes	No	Yes	Other	Yes	Other	Other	No	Other	Yes	Yes	Yes	Yes
4. Were all the subjects selected or recruited from the same or similar populations (including the same time period)? Were inclusion and exclusion criteria for being in the study prespecified and applied uniformly to all participants?	No	Yes	Yes	Yes	Yes	Yes	Yes	Yes	Yes	Yes	Yes	Yes	Yes	Yes	Yes	Yes	Yes
5. Was a sample size justification, power description, or variance and effect estimates provided?	No	No	No	No	No	No	No	No	No	No	No	No	No	No	No	Yes	Yes
6. For the analyses in this paper, were the exposure(s) of interest measured prior to the outcome(s) being measured?	No	Yes	No	No	No	Yes	No	No	No	No	No	No	Other	No	No	No	No
7. Was the timeframe sufficient so that one could reasonably expect to see an association between exposure and outcome if it existed?	No	Yes	No	No	No	Yes	No	No	No	No	No	No	Yes	No	No	No	No
8. For exposures that can vary in amount or level, did the study examine different levels of the exposure as related to the outcome (e.g., categories of exposure, or exposure measured as continuous variable)?	Yes	Yes	Yes	Yes	Yes	Yes	Yes	Yes	Yes	Yes	Yes	Yes	Yes	Yes	Yes	Yes	Yes
9. Were the exposure measures (independent variables) clearly defined, valid, reliable, and implemented consistently across all study participants?	Yes	Yes	Yes	Yes	Yes	Yes	Yes	Yes	Yes	Yes	Yes	Yes	Yes	Yes	Yes	Yes	Yes
10. Was the exposure(s) assessed more than once over time?	No	No	No	No	No	No	No	No	No	No	No	No	No	No	No	No	No
11. Were the outcome measures (dependent variables) clearly defined, valid, reliable, and implemented consistently across all study participants?	Yes	Yes	Yes	Yes	Yes	Yes	Yes	Yes	Yes	Yes	Yes	Yes	Yes	Yes	Yes	Yes	Yes
12. Were the outcome assessors blinded to the exposure status of participants?	Other	Other	Other	Other	Other	Other	Other	Other	Other	Other	Other	Other	Other	Other	Other	Other	Other
13. Was loss to follow-up after baseline 20% or less?	Other	Other	Other	Other	Other	No	Other	Other	Other	Other	Other	Other	Other	Other	Other	Other	Other
14. Were key potential confounding variables measured and adjusted statistically for their impact on the relationship between exposure(s) and outcome(s)?	Yes	Yes	Yes	Yes	Yes	Yes	Yes	Yes	Yes	Yes	Yes	Yes	Yes	Yes	Yes	Yes	Yes
Totals	7	9	8	7	7	9	8	7	8	7	7	7	8	8	8	9	9

	Fung et al. (2022)	Garcia- Hermoso et al.	Gilchrist et al., (2021)	Hajo et al., (2020)	Hinkley et al., (2020)	Hofman et al., (2022)	Hou et al., (2023)	Janssen et al., (2017)	Kandola et al., (2021)	Kitano et al., (2020)	Kuzik et al., (2020)	Kuzik et al., (2022)	Larisch et al., (2020)	Lee et al., (2018)	Le et al., (2021)	Liang et al., (2021)
1. Was the research question or objective in this paper clearly stated?	Yes	Yes	Yes	Yes	Yes	Yes	Yes	Yes	Yes	Yes	Yes	Yes	Yes	Yes	Yes	Yes
2. Was the study population clearly specified and defined?	Yes	Yes	Yes	Yes	Yes	Yes	Yes	Yes	Yes	Yes	Yes	Yes	Yes	Yes	Yes	Yes
3. Was the participation rate of eligible persons at least 50%?	Yes	No	Yes	Yes	Yes	Yes	Other	Yes	No	Yes	Other	Other	No	Yes	Other	Yes
4. Were all the subjects selected or recruited from the same or similar populations (including the same time period)? Were inclusion and exclusion criteria for being in the study prespecified and applied uniformly to all participants?	Yes	Yes	Yes	Yes	Yes	Yes	Yes	Yes	Yes	Yes	Yes	Yes	Yes	Yes	No	Yes
5. Was a sample size justification, power description, or variance and effect estimates provided?	No	No	No	No	No	No	No	No	Yes	No	No	No	No	No	No	No
6. For the analyses in this paper, were the exposure(s) of interest measured prior to the outcome(s) being measured?	Yes	Yes	No	No	Yes	No	No	No	Yes	No	No	No	No	No	No	No
7. Was the timeframe sufficient so that one could reasonably expect to see an association between exposure and outcome if it existed?	Yes	Yes	No	No	Yes	No	no	No	Yes	No	No	No	No	No	No	No
8. For exposures that can vary in amount or level, did the study examine different levels of the exposure as related to the outcome (e.g., categories of exposure, or exposure measured as continuous variable)?	Yes	Other	Yes	Yes	Yes	Yes	Yes	Yes	Yes	Yes	Yes	Yes	Yes	Yes	Yes	Yes
9. Were the exposure measures (independent variables) clearly defined, valid, reliable, and implemented consistently across all study participants?	Yes	Yes	Yes	Yes	Yes	Yes	Yes	Yes	Yes	Yes	Yes	Yes	Yes	Yes	Yes	Yes
10. Was the exposure(s) assessed more than once over time?	Yes	Yes	No	No	No	No	No	No	No	No	No	No	No	No	No	No
11. Were the outcome measures (dependent variables) clearly defined, valid, reliable, and implemented consistently across all study participants?	Yes	Yes	Yes	Yes	Yes	Yes	Yes	Yes	Yes	Yes	Yes	Yes	Yes	Yes	Yes	Yes
12. Were the outcome assessors blinded to the exposure status of participants?	Other	Other	Other	Other	Other	Other	Other	Other	Other	Other	Other	Other	Other	Other	Other	Other
13. Was loss to follow-up after baseline 20% or less?	Yes	No	Other	Other	No	Other	Other	Other	Other	Other	Other	Other	Other	Other	Yes	Other
14. Were key potential confounding variables measured and adjusted statistically for their impact on the relationship between exposure(s) and outcome(s)?	Yes	Yes	Yes	Yes	Yes	Yes	Yes	Yes	Yes	Yes	Yes	Yes	Yes	Yes	Yes	Yes
Totals	12	9	8	8	10	8	7	8	10	8	7	7	7	8	7	8

	Liang et al. (2023)	Li et al., (2022)	Liu et al., (2022)	López-Gil et al., (2022)	Luo et al., (2022)	Luo et al., (2023)	Lu et al., (2021)	McNeill et al., (2020)	Meyer et al., (2020)	Ohta et al., (2023)	Peralta et al., (2022)	Perez et al., (2022)	Porter et al., (2023)	Sampasa- Kanyinga et al., (2020)	Sampasa- Kanyinga et al., (2021)	Sampasa- Kanyinga et al., (2021) PLoS
1. Was the research question or objective in this paper clearly stated?	Yes	Yes	Yes	Yes	Yes	Yes	Yes	Yes	Yes	Yes	Yes	Yes	Yes	Yes	Yes	Yes
2. Was the study population clearly specified and defined?	Yes	Yes	Yes	Yes	Yes	Yes	Yes	Yes	Yes	Yes	Yes	Yes	Yes	Yes	Yes	Yes
3. Was the participation rate of eligible persons at least 50%?	Yes	Yes	Other	Yes	Yes	Yes	Yes	No	Yes	No	Yes	No	No	Yes	Yes	Yes
4. Were all the subjects selected or recruited from the same or similar populations (including the same time period)? Were inclusion and exclusion criteria for being in the study prespecified and applied uniformly to all participants?	Yes	Yes	Yes	Yes	Yes	Yes	Yes	Yes	Yes	Yes	No	Yes	Yes	Yes	Yes	Yes
5. Was a sample size justification, power description, or variance and effect estimates provided?	No	No	No	No	No	No	Yes	No	No	No	No	No	No	No	No	No
6. For the analyses in this paper, were the exposure(s) of interest measured prior to the outcome(s) being measured?	No	No	No	No	No	No	No	Yes	Yes	No	Yes	No	No	No	No	Yes
7. Was the timeframe sufficient so that one could reasonably expect to see an association between exposure and outcome if it existed?	No	No	No	No	No	No	No	Yes	Yes	No	Yes	No	No	No	No	Yes
8. For exposures that can vary in amount or level, did the study examine different levels of the exposure as related to the outcome (e.g., categories of exposure, or exposure measured as continuous variable)?	Yes	Yes	No	Yes	Yes	Yes	Yes	Yes	No	Yes	Yes	Yes	Yes	Yes	Yes	Yes
9. Were the exposure measures (independent variables) clearly defined, valid, reliable, and implemented consistently across all study participants?	Yes	Yes	Yes	Yes	Yes	Yes	Yes	Yes	Yes	Yes	Yes	No	Yes	Yes	Yes	Yes
10. Was the exposure(s) assessed more than once over time?	No	No	No	No	No	No	No	No	No	No	Yes	No	No	No	No	No
11. Were the outcome measures (dependent variables) clearly defined, valid, reliable, and implemented consistently across all study participants?	Yes	Yes	Yes	Yes	Yes	Yes	Yes	Yes	Yes	Yes	Yes	No	Yes	Yes	Yes	Yes
12. Were the outcome assessors blinded to the exposure status of participants?	Other	Other	Other	Other	Other	Other	Other	Other	Other	Other	Other	Other	Other	Other	Other	Other
13. Was loss to follow-up after baseline 20% or less?	Other	Other	Other	Other	Other	Other	Other	No	Yes	Other	No	Other	Other	Other	Other	No
14. Were key potential confounding variables measured and adjusted statistically for their impact on the relationship between exposure(s) and outcome(s)?	Yes	Yes	Yes	Yes	Yes	Yes	Yes	Yes	Yes	Yes	Yes	Yes	Yes	Yes	Yes	Yes
Totals	8	8	6	8	8	8	9	9	10	7	10	5	7	8	8	10

	Sampasa- Kanyinga et al., (2021) JoAH	Sampasa- Kanyinga et al., (2022)	Sampasa- Kanyinga et al., (2022)	St. Laurent et al., (2023)	Taylor et al., (2021)	Taylor et al., (2023)	Vanderlined et al., (2023)	Wang et al., (2022)	Zhang et al., (2022)	Zhang et al., (2023)	Zhu et al., (2019)	Zhu et al., (2023)
1. Was the research question or objective in this paper clearly stated?	Yes	Yes	Yes	Yes	Yes	Yes	Yes	Yes	Yes	Yes	Yes	Yes
2. Was the study population clearly specified and defined?	Yes	Yes	Yes	yes	Yes	Yes	Yes	Yes	Yes	Yes	Yes	Yes
3. Was the participation rate of eligible persons at least 50%?	Yes	Yes	Yes	Other	No	No	Other	No	Yes	Other	No	Yes
4. Were all the subjects selected or recruited from the same or similar populations (including the same time period)? Were inclusion and exclusion criteria for being in the study prespecified and applied uniformly to all participants?	Yes	Yes	Yes	Yes	Yes	Yes	Yes	Yes	Yes	Yes	Yes	Yes
5. Was a sample size justification, power description, or variance and effect estimates provided?	No	No	No	No	No	No	No	No	No	No	No	No
6. For the analyses in this paper, were the exposure(s) of interest measured prior to the outcome(s) being measured?	No	No	No	No	Yes	Yes	No	No	No	Yes	No	No
7. Was the timeframe sufficient so that one could reasonably expect to see an association between exposure and outcome if it existed?	No	No	No	No	Yes	Yes	No	No	No	Yes	No	No
8. For exposures that can vary in amount or level, did the study examine different levels of the exposure as related to the outcome (e.g., categories of exposure, or exposure measured as continuous variable)?	Yes	Yes	Yes	Yes	No	Yes	Yes	Yes	Yes	Yes	Yes	Yes
9. Were the exposure measures (independent variables) clearly defined, valid, reliable, and implemented consistently across all study participants?	Yes	Yes	Yes	Yes	Yes	Yes	Yes	Yes	Yes	Yes	Yes	Yes
10. Was the exposure(s) assessed more than once over time?	No	No	No	No	Yes	Yes	No	No	No	No	No	No
11. Were the outcome measures (dependent variables) clearly defined, valid, reliable, and implemented consistently across all study participants?	Yes	Yes	Yes	yes	Yes	Yes	Yes	Yes	Yes	Yes	Yes	Yes
12. Were the outcome assessors blinded to the exposure status of participants?	Other	Other	Other	Other	Other	Yes	Other	Other	Other	Other	Other	Other
13. Was loss to follow-up after baseline 20% or less?	Other	Other	Other	Other	No	Yes	Other	Other	Other	No	Other	Other
14. Were key potential confounding variables measured and adjusted statistically for their impact on the relationship between exposure(s) and outcome(s)?	Yes	Yes	Yes	Yes	Yes	Yes	Yes	Yes	Yes	Yes	Yes	Yes
Totals	8	8	8	7	9	11	7	7	8	9	7	8

## Discussion

This systematic review synthesized the evidence surrounding the relationships between combinations of 24-h movement behaviors—physical activity, sleep, and sedentary behaviors—and a range of different indicators of mental ill-being and well-being across the lifespan. A total of 597,705 participants from 16 different countries were represented in the 73 studies that met our inclusion criteria. The majority of studies included samples of children and youth (*n* = 47), though roughly one third of the studies focused on adults (*n* = 26). The volume of studies involving adult samples is promising given 24-h movement guidelines specific to these age groups were only released in 2020 [39]. Although more longitudinal work has been published in recent years (*n* = 15), the body of literature largely consists of cross-sectional studies (*n* = 58). Different research questions related to the associations between movement behaviors and indicators of mental health have been quantified using seven different analytical approaches, with the vast majority of studies investigating total or specific combinations of guideline adherence, followed by compositional data analysis, isotemporal substitution, latent profile or cluster-based analysis, the Goldilocks method, and rest-activity rhythmicity. The diversity of analytical methods employed in this literature highlights the complexity of quantifying these associations but converges on a critical consensus: engaging in a healthy balance of movement behaviors characterized by accruing adequate sleep, maximizing MVPA, and minimizing sedentary behaviors, regardless of age, appears to be beneficial for mental health. However, the need for more longitudinal studies and device-based measurement to improve the precision in our estimates remains to fully understand the implications of movement behaviors on mental health and to tailor recommendations that can effectively promote mental well-being and reduce ill-being across the lifespan.

At present, cross-sectional studies investigating guideline adherence, whether it be total guidelines met or specific combinations, with self- or proxy-reported measures remain dominant. From a behavioral surveillance standpoint this is not surprising given that several countries have independent recommendations for each movement behavior irrespective of whether they have adopted integrated 24-h guidelines or not, and their surveillance systems have allowed researchers to investigate the collective effects of physical activity, sedentary behavior, and sleep on outcomes such as mental health. While monitoring guideline adherence is important for the public health goal of promoting well-being in the population, the threshold-based nature of these guidelines can be limiting. Specifically, cut-point criterions neglect the dose–response nature of the relationships that have been observed between each movement behavior and indicators of mental health (e.g., [17, 65, 66]) and the co-dependent nature of 24-h movement behaviors. Nevertheless, our synthesis of the evidence generally found that adhering to an increasing number of the three guidelines was associated with more favorable scores for several indicators of mental ill-being and well-being across the lifespan, including some evidence supporting a dose–response gradient [62, 67–73]. While null effects were observed in several studies, over half (63% for children/youth; 62% for adults) of the cross-sectional associations, and both of the longitudinal associations for adults included in this review provided support for continuing to promote an integrated 24-h approach that targets all three behaviors. It should be noted that weakest among the evidence supporting a link between movement behaviors and mental health was the longitudinal work conducted among children and youth. One potential reason for these findings is that most of these associations were derived from studies of young children [74, 75]. Given that early childhood precedes life stages when mental health problems become more apparent (e.g., adolescence) [76], there may not be enough variance within the indicators of mental health to observe significant associations. Previous research investigating cross-sectional associations between wake-time movement compositions and mental health among preschool children supports this notion [77]. Moving forward, studies examining guideline adherence may provide a simple barometer of the importance of 24-h movement behaviors for mental health, although it should be recognized that the use of alternative approaches such as compositional data analysis may provide a more nuanced and precise understanding of these relationships.

With the prevailing shift to the 24-h movement paradigm, there has also been an emphasis on adopting device-based measures that can capture movement across the whole day. Although not without limitations, accelerometry provides an opportunity to use compositional data analysis, and therefore considers the co-dependent nature of 24-h movement behavior data. Our review found accelerometry was used most often to capture physical activity data (42% of studies), followed by sedentary behavior (32% of studies), and sleep (27% of studies). The range in usage across behaviors is interesting, but likely reflects the fact that accelerometry cannot capture recreational screen time (i.e., to assess the sedentary component of the 24-h guidelines). Additionally, protocols with hip-worn devices can be uncomfortable for participants to sleep with. Despite this disparity, a total of 19 studies (26% of those included) used compositional data analysis techniques, including 14 studies that used compositional isotemporal substitution, two studies that used the novel Goldilocks approach to identify a combination of movement behaviors that optimizes mental health, and one study that investigated rest-activity rhythmicity. This represents considerable growth since Sampasa-Kanyinga et al.'s [44] systematic review of children and youth, which identified only two studies that used compositional data analysis. The increased application of compositional data analysis techniques is indicative of its increase in popularity and uptake by researchers investigating the relationships between 24-h movement behaviors and indicators of mental health. Findings from these analyses, however, reveal disparities across the age groups and study designs to date. Specifically, 55% of the cross-sectional associations from studies of children and youth observed a significant association between the 24-h movement composition and mental health compared to only 31% for adults, with similar findings across the indicators of mental ill-being and well-being for both groups. Further, none of the nine longitudinal studies demonstrated significant associations between the 24-h movement composition at baseline and mental health at follow-up. It is worth noting that the strength of the composition-based literature is likely underestimated as several manuscripts have reported significant effects for individual behaviors within the 24-h composition (relative to others) or isotemporal substitutions (reallocating sedentary behavior to sleep or MVPA, in particular) but have overlooked reporting the full 24-h composition model. Authors of these studies suggest some of the null findings may be due to small sample sizes (i.e., limited power to detect significant effects) or limited changes in the mental health outcomes at follow up [78, 79]. Another possible explanation relates to a limitation of using devices in that they are unable to capture contextual information. The setting in which physical activity takes place (e.g., occupational versus leisure time), and the type of sedentary behavior (e.g., having coffee with a friend versus scrolling social media) could have vastly different impacts on mental health [20, 30]. Future studies that pair wearable devices with signal-contingent ecological momentary assessments are warranted as they stand to provide information related to other aspects of these behaviors beyond time-based estimates of behavioral engagement. Given the potential for improving our understanding of the relationships between 24-h movement behaviors and mental health, an emphasis should be placed on leveraging data from wearable devices as they continue to become more ubiquitous in commercial and medical use. Although strict data protection policies need to be in place, longitudinal data derived from these devices could be used in various compositional models for the purpose of improving our understanding of the causal nature of these relationships.

As previously mentioned, given that 24-h movement guidelines for children and youth guidelines were first released, there was a considerably smaller evidence base focusing on adult populations. Despite this disparity, differences and similarities in the patterns of results across the age groups deserve attention. First, adhering to all three guidelines was consistently found to most often be correlated with favorable benefits for mental health compared to meeting fewer guidelines for both children and youth (63%) as well as for adults (62%). Closer inspection of these results revealed that meeting all three guidelines was more consistently associated with indicators of mental well-being among children and youth (79% of associations versus 54% for ill-being), whereas the opposite was found for adults (71% of associations for mental ill-being versus 43% for well-being). However, considering the heterogeneity in indicators of mental health assessed, in addition to the difference in the size of the samples these inferences were drawn from, these findings should be interpreted with caution. Second, a considerable disparity was observed within the isotemporal substitution analyses wherein children and youth more consistently experience mental health benefits from replacing sedentary behavior or recreational screen time with either MVPA (82% for children and youth versus 47% for adults) or sleep (76% for children and youth versus 35% for adults), although it is important to note that the patterns of results were consistent across indicators of mental ill-being and well-being for both groups. One potential explanation for this finding could be that sedentary pursuits such as social media engagement may have a greater influence on mental health among children and youth. Specifically, harmful online feedback from peers may have a greater adverse impact on mental health during childhood and adolescence as the process of identity formation unfolds before a more fixed sense of self is recognized in adulthood [80]. Children and youth may also be more likely to struggle with regulating their emotional responses to such interactions given their prefrontal cortex has yet to mature [81]. Overall, evidence for both of these populations shows some coherence but it’s clear that more longitudinal research is needed to improve our understanding of the nature these relationships so that we can identify specific life stages when certain movement behaviors may confer the greatest benefits for mental health.

As emerging research continues to advance knowledge regarding the impacts of 24-h movement behaviors on mental health, it is clear that there are several different analytic approaches that each provides unique insight into this relationship. Studies examining guideline adherence will likely remain the foundation of this body of evidence as population-level behavioral surveillance systems continue to monitor the prevalence of 24-h guideline adherence using self- and proxy-reported surveys or a mix of self-reports and device-measured behavior. Beyond guideline adherence, the field is strongly encouraged to continue adopting compositional data analysis techniques given their appropriateness for analyzing time-use data within a fixed window (i.e., 24 h). The evidence base linking movement compositions to indicators of mental health will inevitably continue to develop in the coming years, yet findings from isotemporal studies make it clear that reallocating time from sedentary behaviors to sleep or physical activity should be prioritized in the meantime. New compositional techniques will continue to provide important insights into these relationships. For example, application of the Goldilocks method [82] has provided seminal knowledge regarding the optimal daily movement composition for indicators of mental health, whereas other emerging techniques such as the “Many roads lead to Rome” [83] approach have yet to be applied to mental health data yet but will yield important information regarding different iterations of movement compositions that can provide equivalently optimal benefits when eventually implemented. Similar to the Goldilocks method, rest-activity rhythmicity has received limited application to date but represents an excellent opportunity to understand how within- and between-day variability in sleep and movement may influence mental health. Finally, latent profile and cluster-based techniques should be considered for the purpose of identifying unique groups in the population that may stand to benefit most from targeted and tailored interventions [84]. Existing evidence has made it clear that a small fraction of the population engages in the least healthy combination of movement behaviors and membership in this group corresponds with the poorest scores on measures of mental health [53, 57, 85], which underscores the importance of allocating more public health resources to these individuals. In sum, this review has highlighted that there is currently a plethora of analytical tools at the disposal of researchers attempting to improve our current understanding of the relationship between 24-h movement behaviors and mental health, but novel techniques are emerging rapidly and should receive full consideration for the purpose of continuing to advance knowledge in this field.

With the 24-h movement paradigm receiving increasing interest as an integrative approach to promote mental well-being and prevent or manage mental ill-being, new evidence is needed to gain further insights into the interplay between the mechanisms underlying these relationships. The mechanisms that link physical activity, sedentary behaviors, and sleep with mental health are understandably complex and multifaceted, reflecting interactions across biological, psychosocial, and behavioral domains. Independent bodies of literature have provided a foundation of knowledge related to mechanisms by which each movement behavior influences indicators of mental health thus far. Sedentary behaviors, for example, have been associated with unfavorable effects on indicators of mental health via increased exposure to stressors such as negative social comparisons or cyberbullying [86–88], although it is important to recognize that these mechanistic pathways may be specific to recreational screen time and social media use as opposed to simply engaging in low energy expenditure behaviors. Excess sedentary behavior has also been linked to a higher likelihood of experiencing sleep disorders and disruptions [89], and the displacement of time that could be spent on physically or socially engaging activities that confer mental health benefits [90, 91]. Our isotemporal substitution findings align with this mechanistic understanding in that replacing sedentary behaviors with physical activity or sleep was found to have beneficial effects for several indicators of mental health. The mechanisms by which physical activity impacts mental health have received considerable attention to date. Specifically, biological (e.g., changes in the structural and functional composition of the brain, reduced inflammation and oxidative stress), psychosocial (e.g., social interactions, self-perceptions, interactions with the natural environment), and behavioral factors (e.g., improved sleep quantity and quality, development of self-regulatory and coping skills) have been identified as key intermediary mechanisms [92, 93]. Finally, adequate sleep has been to linked mental health—emotional regulation, depression, anxiety, and stress-related disorders in particular—via reducing cellular inflammation, stimulating or attenuating brain neurotransmitter activity (e.g., via the adenosinergic receptor system), and optimizing hypothalamic–pituitary–adrenal axis functioning, which plays a role in stress reactivity [94–98]. While these examples of mechanistic pathways are not exhaustive by any means, the overarching focus on how time-based estimates of movement behaviors relate to indicators of mental health obfuscates the impact of other relevant aspects of these behaviors such as quality-based (e.g., sleep quality, quality physical activity participation experiences [99, 100] and contextual factors (e.g., physical activity setting, sedentary behavior domains [20, 30]. Capturing data about these other important aspects of movement behaviors will help to elucidate the mechanisms underlying relationships with indicators of mental health. Doing so is critical for informing the development of tailored interventions that promote the optimal aspects of 24-h movement behaviors collectively to address the mental health issues facing society today.

Despite addressing an existing knowledge gap, this review is not without limitations. First, most studies have used cross-sectional designs which limits our ability to make causal inferences. While the recency of existing 24-h movement guidelines has certainly contributed to the lack of longitudinal work, researchers are encouraged to inspect older data sources as they may have included measures needed to investigate how combinations of movement behaviors influence mental health over time. Second, only studies published in English were included in the present review. We recognize that studies published in languages other than English may exist, but what was included in our search likely represents the greater majority of the literature published to date. Third, across the studies there was considerable variety in not only the tools employed to assess each movement behavior, but also in the measures of mental health outcomes. Although studies included measures that have shown strong psychometric properties, heterogeneity in the assessment tools used complicates any comprehensive synthesis of the literature.

In sum, this review was the first study to synthesize the evidence examining how physical activity, sleep and sedentary behaviors collectively relate to indicators of mental ill-being and well-being across the lifespan. Taken together, and with the emergence of several advanced analytical approaches, findings reinforce the notion that time spent engaging in physical activity and sleep need to be prioritized for promoting mental health and well-being, especially when replacing sedentary pursuits such as recreational screen time. This is an emerging literature—for adults in particular—and more longitudinal work is required to make stronger inferences. Nevertheless, moving forward public health messaging should continue to shift away from a siloed approach focused on individual behaviors to instead emphasize that the whole day counts when it comes to the importance of movement for mental health and well-being.

## Supplementary Information

**Additional file 1**. PRISMA Checklist and Abstract Checklist.

**Additional file 2.** Search terms.

**Additional file 3.** Table by associations.

## Data Availability

Not applicable.
