# Supplementary material for: Associations between 24-h movement behaviors and indicators of mental health and well-being across the lifespan: a systematic review
Source: J Act Sedentary Sleep Behav. 2024 Mar 14;3:9. doi: 10.1186/s44167-024-00048-6 (PMC11960375; doi:10.1186/s44167-024-00048-6)
Supplement: Supplementary file 2 — Additional file 2. Search terms. [file 44167_2024_48_MOESM2_ESM.docx]

**Additional file 2**. *Search Terms*

# Keywords – Exercise AND Sleep AND Sedentary behavior

## MEDLINE (EBSCO)

(MH "Exercise+") OR (MH "Exercise Movement Techniques+") OR (MH "Exercise Therapy+") OR (MH "Sports+") OR (MM "Physical Exertion") OR (MM "Physical Education and Training") OR sports OR (sport* OR bicycl* OR swim* OR walk* OR run* OR jog*) OR (physical w2 activity) OR (aerobic w2 (train* OR activ*)) OR (activ* n2 outdoor*) OR (activ* w3 (space* OR behavi#r* OR transport* OR commut* OR neighbo#rhood* OR park* OR game* OR gaming OR lifestyle)

AND

Sleep NOT ((MH "Sleep Apnea Syndromes+") OR (TI (Apnea OR apnoea)))

AND

(sedentary lifestyle) OR TI (bed rest OR (sedentary OR inactiv* OR sitting OR (lack w2 activity) OR (low w4 energy expend*)) OR AB ((bed rest OR (sedentary OR inactiv* OR sitting OR (lack w2 activity) OR (low w4 energy expend*)) OR ((chair OR car OR automobil* OR auto OR bus OR (motor vehicle*) OR indoor* OR in-door* OR screen OR computer) w3 time) OR (MH "Motion Pictures") OR (MH "Video Games+") OR (MH "Mass Media+") OR (MM "Cartoons as Topic") OR (MH "Computers, Handheld+") OR (MH "Computers+") OR (MH "Software+") OR (MH "Video Recording+") OR (MH "Social Media") OR (MM "Webcasts as Topic") OR (MH "Communications Media") OR (MH "Videodisc Recording+") OR (TI (television OR tv OR screentime OR ((watch* OR view*) w2 (dvd* OR video*)) OR (screen media) OR (social media) OR (video gam*) OR videogam* OR (computer gam*) OR (electronic gam*)) OR AB (television OR tv OR screentime OR ((watch* OR view*) w2 (dvd* OR video*)) OR (screen media( OR (social media) OR (video gam*) OR videogam* OR (computer gam*) OR (electronic gam*))) OR (TI ((screen based entertainment) OR (screen-based entertainment) OR smartphone* OR ipad OR apps OR app OR (mobile applications)) OR ab ((screen based entertainment) OR (screen-based entertainment) OR smartphone* OR ipad OR apps OR app OR (mobile applications))

Limited to: 2009-current, English, Scholarly (Peer-reviewed) Journals, Academic Journals

## EMBASE (Ovid)

(exp physical activity/ OR exp exercise/ OR exp kinesiotherapy/ OR physical education/ OR exp sport/) OR ((sport* OR bicycl* OR swim* OR walk* OR run* OR jog*).tw,kw.) OR ((aerobic adj2 (train$ OR active$)).tw,kw.) OR (((activ* OR outdoor*) adj3 play*).tw,kw.) OR (active.ti. and (space* OR behavio?r* OR transport* OR commut* OR neighbo?rhood* OR park* OR game* OR gaming OR lifestyle).mp.) OR ((active adj3 (space* OR behavio?r* OR transport* OR commut* OR neighbo?rhood* OR park* OR game* OR gaming OR lifestyle)).tw,kw.)

AND

(Sleep/) OR (Sleep Time/) OR ((sleep adj3 duration).tw.) NOT (exp Sleep Disordered Breathing/ and (apnea OR apnoea).ti.)

AND

(sedentary lifestyle/) OR (bed rest.mp. OR (sedentary OR inactiv* OR sitting OR (lack adj2 activity) OR (low adj3 energy expend*)).ti,ab,kw.) OR (((chair OR car OR automobile* OR auto OR bus OR motor vehicle* OR indoor* OR in-door* OR screen OR computer) adj time).ti,ab,kw.) OR (video game/ OR software/ OR videorecording/ OR movie/ OR exp mass communication/ OR television viewing/ OR television/ OR exp computer/ OR internet addiction/ OR mobile application/ OR exp mobile phone/) OR ((television OR tv OR screentime OR ((watch* OR view*) adj2 (dvd* OR video*)) OR screen media OR social media OR video gam* OR videogam* OR computer gam* OR electronic gam*).ti,ab,kw.) OR ((screen based entertainment OR screen-based entertainment OR smartphone* OR ipad OR apps OR app OR mobile applications).ti,ab,kw.)

Limit to (embase and (english) and yr="2009 -Current")

## PsycINFO (EBSCO)

(MM "Exercise" OR MM "Aerobic Exercise" OR MM "Weightlifting" OR MM "Yoga") OR (MM "Sports" OR MM "Adaptive Sports" OR MM "Athletes" OR MM "Athletic Participation" OR MM "Athletic Performance" OR MM "Baseball" OR MM "Basketball" OR MM "College Sports" OR MM "Cycling" OR MM "Extreme Sports" OR MM "Football" OR MM "High School Sports" OR MM "Judo" OR MM "Martial Arts" OR MM "Professional Sports" OR MM "Soccer" OR MM "Swimming" OR MM "Tennis" OR MM "Weightlifting") OR (MM "Physical Education") OR (MM "Physical Activity" OR MM "Actigraphy") OR (MM "Activity Level") OR (MM "Movement Therapy") OR (MM "Dance Therapy") OR (MM "Energy Expenditure") OR (MM "Mind Body Therapy") OR (sport* OR bicycl* OR swim* OR walk* OR run* OR jog*) OR (physical w2 activity) OR (aerobic w2 (train* OR activ*) OR (activ* n2 outdoor*) OR (activ* w3 (space* OR behavi#r* OR transport* OR commut* OR neighbo#rhood* OR park* OR game* OR gaming OR lifestyle)

AND

((MM "Sleep" OR MM "Dreaming" OR MM "Napping" OR MM "NREM Sleep" OR MM "REM Sleep" OR MM "Sleep Onset" OR MM "Sleep Quality" OR MM "Snoring")) OR (MM "Sleep Deprivation") OR TI(sleep n3 duration) OR AB(sleep n3 duration) NOT (DE "Sleep Apnea") OR (TI (apnea OR apnoea)))

AND

(MM "Sedentary Behavior") OR (sedentary lifestyle) OR TI (bed rest OR (sedentary OR inactiv* OR sitting OR (lack w2 activity) OR (low w4 energy expend*)) OR AB ((bed rest OR (sedentary OR inactiv* OR sitting OR (lack w2 activity) OR (low w4 energy expend*)) OR ((chair OR car OR automobil* OR auto OR bus OR (motor vehicle*) OR indoor* OR in-door* OR screen OR computer) w3 time) OR (MM "Screen Time") OR (MM "Television Viewing") OR (MM "Computers" OR MM "Analog Computers" OR MM "Cloud Computing" OR MM "Computer Games" OR MM "Computer Peripheral Devices" OR MM "Computer Software" OR MM "Digital Computers" OR MM "Microcomputers" OR MM "Mobile Devices") OR (MM "Computer Usage" OR MM "Internet Usage" OR MM "Online Behavior") OR (MM "Computer Games") OR (MM "Digital Gaming") AND (MM "Internet" OR MM "Blog" OR MM "Internet Addiction" OR MM "Internet Usage" OR MM "Digital Gaming" OR MM "Online Dating" OR MM "Online Social Networks" OR MM "Smartphone Use") OR (MM "Communications Media" OR MM "Audiovisual Communications Media" OR MM "Digital Media" OR MM "Mass Media" OR MM "Multimedia" OR MM "Social Media" OR MM "Telecommunications Media") OR (MM "Mobile Devices" OR MM "Mobile Phones" OR MM "Tablet Computers") OR (MM "Mobile Applications") AND (MM "Smartphone Use" OR MM "Smartphones") OR (MM "Television" OR MM "Closed Circuit Television" OR MM "Educational Television" OR MM "Television Advertising") OR (MM "Films") OR (MM "Mass Media" OR MM "Films" OR MM "News Media" OR MM "Printed Communications Media" OR MM "Radio" OR MM "Television") OR (MM "Cartoons (Humor)") OR (MM "Social Media" OR MM "Online Social Networks") OR (TI (television OR tv OR screentime OR ((watch* OR view*) w2 (dvd* OR video*)) OR (screen media) OR (social media) OR (video gam*) OR videogam* OR (computer gam*) OR (electronic gam*)) OR AB (television OR tv OR screentime OR ((watch* OR view*) w2 (dvd* OR video*)) OR (screen media( OR (social media) OR (video gam*) OR videogam* OR (computer gam*) OR (electronic gam*))) OR (TI ((screen based entertainment) OR (screen-based entertainment) OR smartphone* OR ipad OR apps OR app OR (mobile applications)) OR ab ((screen based entertainment) OR (screen-based entertainment) OR smartphone* OR ipad OR apps OR app OR (mobile applications))

Limited to: 2009-Current, English, Academic Journals

## SportDISCUS (EBSCO)

((DE "EXERCISE" OR DE "ABDOMINAL exercises" OR DE "AEROBIC exercises" OR DE "ANAEROBIC exercises" OR DE "AQUATIC exercises" OR DE "ARM exercises" OR DE "BACK exercises" OR DE "BREATHING exercises" OR DE "BREEMA" OR DE "BUTTOCKS exercises" OR DE "CALISTHENICS" OR DE "CHAIR exercises" OR DE "CHEST exercises" OR DE "CIRCUIT training" OR DE "COMPOUND exercises" OR DE "COOLDOWN" OR DE "DO-in" OR DE "EXERCISE adherence" OR DE "EXERCISE for children" OR DE "EXERCISE for girls" OR DE "EXERCISE for men" OR DE "EXERCISE for middle-aged persons" OR DE "EXERCISE for older people" OR DE "EXERCISE for people with disabilities" OR DE "EXERCISE for women" OR DE "EXERCISE for youth" OR DE "EXERCISE therapy" OR DE "EXERCISE video games" OR DE "FACIAL exercises" OR DE "FALUN gong exercises" OR DE "FOOT exercises" OR DE "GYMNASTICS" OR DE "HAND exercises" OR DE "HATHA yoga" OR DE "HIP exercises" OR DE "ISOKINETIC exercise" OR DE "ISOLATION exercises" OR DE "ISOMETRIC exercise" OR DE "ISOTONIC exercise" OR DE "KNEE exercises" OR DE "LEG exercises" OR DE "LIANGONG" OR DE "METABOLIC equivalent" OR DE "MULAN quan" OR DE "MUSCLE strength" OR DE "PILATES method" OR DE "PLYOMETRICS" OR DE "QI gong" OR DE "REDUCING exercises" OR DE "RUNNING" OR DE "RUNNING -- Social aspects" OR DE "SCHOOL exercises & recreations" OR DE "SEXUAL exercises" OR DE "SHOULDER exercises" OR DE "STRENGTH training" OR DE "STRESS management exercises" OR DE "TAI chi" OR DE "TREADMILL exercise" OR DE "WHEELCHAIR workouts" OR DE "YOGA") AND (DE "SPORTS" OR DE "AERODYNAMICS in sports" OR DE "AERONAUTICAL sports" OR DE "AGE & sports" OR DE "AMATEUR sports" OR DE "ANIMAL sports" OR DE "ANTISEMITISM in sports" OR DE "AQUATIC sports" OR DE "BALL games" OR DE "BALLISTICS in sports" OR DE "BASEBALL" OR DE "BIOMECHANICS in sports" OR DE "COLLEGE sports" OR DE "COMBAT sports" OR DE "COMMUNICATION in sports" OR DE "CONTACT sports" OR DE "CROSS-training (Sports)" OR DE "DISC golf" OR DE "DISCRIMINATION in sports" OR DE "DOG sports" OR DE "DOPING in sports" OR DE "ENDURANCE sports" OR DE "EXTREME sports" OR DE "FANTASY sports" OR DE "FASCISM & sports" OR DE "FEMINISM & sports" OR DE "GAELIC games" OR DE "GAY Games" OR DE "GOODWILL Games" OR DE "GYMNASTICS" OR DE "HOCKEY" OR DE "HOMOPHOBIA in sports" OR DE "HYDRODYNAMICS in sports" OR DE "INDIVIDUAL sports" OR DE "KINEMATICS in sports" OR DE "KNIFE throwing" OR DE "LGBTQ+ people & sports" OR DE "LOG-chopping (Sports)" OR DE "MASCULINITY in sports" OR DE "MASS media & sports" OR DE "MILITARY sports" OR DE "MINORITIES in sports" OR DE "MOTION pictures in sports" OR DE "MOTORSPORTS" OR DE "NATIONAL socialism & sports" OR DE "NATIONALISM & sports" OR DE "NONVERBAL communication in sports" OR DE "OLYMPIC Games" OR DE "PARKOUR" OR DE "PHYSICS in sports" OR DE "PRESIDENTS -- Sports" OR DE "PROFESSIONAL sports" OR DE "PROFESSIONALISM in sports" OR DE "RACISM in sports" OR DE "RACKET games" OR DE "RADAR in sports" OR DE "RECREATIONAL sports" OR DE "REGIONALISM & sports" OR DE "ROBOTICS in sports" OR DE "RODEOS" OR DE "ROLLER skating" OR DE "SCHOOL sports" OR DE "SENIOR Olympics" OR DE "SEXUAL harassment in sports" OR DE "SHOOTING (Sports)" OR DE "SHUTOUTS (Sports)" OR DE "SKATEBOARDING" OR DE "SOCIALISM & sports" OR DE "SOFTBALL" OR DE "SPORT for all" OR DE "SPORTS & state" OR DE "SPORTS & technology" OR DE "SPORTS & theater" OR DE "SPORTS & tourism" OR DE "SPORTS for children" OR DE "SPORTS for girls" OR DE "SPORTS for older people" OR DE "SPORTS for people with disabilities" OR DE "SPORTS for youth" OR DE "SPORTS forecasting" OR DE "SPORTS in antiquity" OR DE "SPORTS penalties" OR DE "SPORTS photography" OR DE "SPORTS rivalries" OR DE "SPORTS teams" OR DE "SPORTS tourism" OR DE "STEREOTYPES in sports" OR DE "TARGETS (Sports)" OR DE "TEAM sports" OR DE "TEAMWORK (Sports)" OR DE "TELEVISION & sports" OR DE "TRACEURS" OR DE "VIDEO tapes in sports" OR DE "VIOLENCE in sports" OR DE "WINTER sports" OR DE "WOMEN'S sports" OR DE "PHYSICAL education" OR DE "COACHING (Athletics)" OR DE "COLLEGE sports" OR DE "DRILLS (Practice)" OR DE "FIELD days (Education)" OR DE "FUNCTIONAL training" OR DE "MOTOR learning" OR DE "MOVEMENT education" OR DE "MUSIC in physical education" OR DE "PHYSICAL Education Attitude Inventory" OR DE "PHYSICAL Education Teacher Assessment Instrument" OR DE "PHYSICAL education (Elementary)" OR DE "PHYSICAL education (Middle school)" OR DE "PHYSICAL education (Primary)" OR DE "PHYSICAL education (Secondary)" OR DE "PHYSICAL education for children" OR DE "PHYSICAL education for girls" OR DE "PHYSICAL education for older people" OR DE "PHYSICAL education for people with disabilities" OR DE "PHYSICAL education for women" OR DE "PLAYGROUND games" OR DE "PRACTICE (Sports)" OR DE "SCHOOL sports" OR DE "SPORTS clinics" OR DE "SPORTS sciences" OR DE "SWEDISH gymnastics" OR DE "SWIMMING for children -- Training" OR DE "TELEVISION in physical education" OR DE "PHYSICAL education (Elementary)" OR DE "PHYSICAL education (Middle school)" OR DE "PHYSICAL education (Primary)" OR DE "PHYSICAL education (Secondary)")) OR (DE "PHYSICAL activity") OR TI ( exercise OR (physical* n2 activ*) OR (aerobic N2 train* OR aerobic N2 active* OR sport* OR outdoor* OR (active N3 recreation*) OR run* OR walk* OR jog* OR bicycl* OR biking OR cycling OR swim* OR soccer OR gymnastic* ) OR SU (exercise OR (physical* n2 activ*) OR aerobic N2 train* OR aerobic N2 active* OR sport* OR outdoor* OR "active recreation*" OR run* OR walk* OR jog* OR bicycl* OR swim* OR (active N3 recreation*))

AND

(DE "SLEEP" OR DE "SLEEP hygiene") NOT (DE "SLEEP apnea syndromes in children") OR Sleep NOT ((TI (apnea OR apnoea)) OR SU (apnea OR apnoea)

AND

(DE "MASS media" OR DE "AGING in mass media" OR DE "ATHLETES in mass media" OR DE "BODY image in mass media" OR DE "MASS media & sports" OR DE "RACE in mass media" OR DE "SURFING in mass media") OR (DE "INTERNET") AND (DE "VIDEO games" OR DE "COMPUTER baseball games" OR DE "DANCE Dance Revolution (Game)" OR DE "EXERCISE video games" OR DE "SPORTS in video games" OR DE "VIDEO games & children" OR DE "VIDEO games & teenagers" OR DE "VIDEO games & children" OR DE "VIDEO games & teenagers" OR DE "VIDEO games -- Physiological aspects") OR (DE "SEDENTARY behavior") OR (DE "SEDENTARY behavior in children") OR (smartphone* OR ipad OR apps OR app OR mobile applications) OR screen based entertainment OR ((television N3 time) OR screentime OR (screen N3 time) OR (computer N3 time) OR ((watch* OR view*) N2 (dvd OR N2 video*)) OR (screen media) OR (social media) OR (video gam*) OR videogam* OR (computer gam*) OR (electronic gam*) OR ((chair OR car OR automobile OR auto OR (motor vehicle) OR bus OR indoor* OR in-door* OR computer) N3 time) OR sitting OR (sedentary OR inactiv* OR (lack N2 activity) OR (low N3 energy expend*) OR (physical* inactiv*)

Limited to: 2009-Current, English, Academic Journals
