# Supplementary material for: Associations between 24-h movement behaviors and indicators of mental health and well-being across the lifespan: a systematic review
Source: J Act Sedentary Sleep Behav. 2024 Mar 14;3:9. doi: 10.1186/s44167-024-00048-6 (PMC11960375; doi:10.1186/s44167-024-00048-6)
Supplement: Supplementary file 3 — Additional file 3. Table by associations. [file 44167_2024_48_MOESM3_ESM.docx]

| Children and Youth |  |  |  |  |  |
| --- | --- | --- | --- | --- | --- |
|  | Cross-sectional |  |  |  |  |
|  |  | Flourishing |  |  |  |
|  |  |  | Total Guidelines |  |  |
|  |  |  |  | 1/1 studies found a favorable dose-response relationship for number of guidelines met | Wang 2022 |
|  |  |  | Combination guidelines | 1/1 studies showed that compared to meeting none of the guidelines, meeting all 3 guidelines were associated with the most favorable scores | Wang 2022 |
|  |  |  |  |  |  |
|  |  |  | Isotemporal Substitution |  |  |
|  |  |  |  | When not meeting the sleep guideline: -2/2 studies showed that replacing screen time with sleep or MVPA was associated with more favorable scores -1/2 studies showed that replacing sleep with MVPA was associated with more favorable scores (Gilchrist, 2021)  When meeting the sleep guideline: -1/2 studies showed that replacing screen time with sleep was associated with more favorable scores (Gilchrist, 2021) -2/2 studies showed that replacing screen time or sleep with MVPA was associated with more favorable scores | Gilchrist 2021; Brown 2021 |
|  |  |  | Latent profiles |  |  |
|  |  |  |  | 1/1 studies showed the the healthiest combination of movement behaviors (adequate sleep, high MVPA, low ST) was associated with the most favorable scores | brown 2021 |
|  |  | Depressive symptoms |  |  |  |
|  |  |  | Total Guidelines |  |  |
|  |  |  |  | 5/8 studies showed that meeting three guidelines was associated with the most favorable scores compared to meeting none of the guidelines (Feng 2022; Liang, 2023; Lu, 2021; Brown, 2021 EB [population norms subsample only, not active/inactive epilepsy subsamples], Zhang 2023) 1/8 studies showed that meeting two of the guidelines was associated with the most favorable scores compared to meeting none of the guidelines (Brown 2021 JPAH) 2/8 studies showed no differences between meeting any number of guidelines compared to meeting none of the guidelines (Brown, 2021 EB; active and inactive epilepsy samples) | Lu 2021; Brown 2021 JPAH; Brown, 2021 EB; Feng 2022; Liang 2023; Zhang 2023 |
|  |  |  | Combinations of Guidelines |  |  |
|  |  |  |  | 1/9 studies showed that meeting the PA+ST guidelines was associated with the most favorable scores compared to meeting none of the guidelines (Feng, 2022) 5/9 studies showed that meeting all 3 guidelines was associated with the most favorable scores compared to meeting none of the guidelines (Liang, 2022; Lu, 2021; Zhang 2023), including among 12-17 year olds (Zhu, 2019) 2/9 studies showed that meeting the SL+ST guidelines was associated with the most favorable scores compared to meeting none of the guidelines (Sampasa-Kanyinga, 2021; Hou 2023) 1/9 studies showed that compared to meeting all 3 guidelines, meeting the PA+SL guidelines was associated with the most favorable scores among 6-11 year olds (Zhu, 2019) | Lu 2021; Sampasa-Kanyinga 2021, Feng, 2022, Liang 2023, Zhu 2019; Hou 2023; Luo 2023; Zhang 2023 |
|  |  |  | Compositional Analysis |  |  |
|  |  |  |  | 1/5 studies found an association with the 24-h movement composition (Dumuid, 2022) | Fairclough 2021; Dumuid 2022; Taylor 2023 |
|  |  |  | Goldilocks Method |  |  |
|  |  |  |  | 1/1 studies found the optimal 24-hr composition was 11.4h of sleep, 8.4h of sedentary time, 2.3h of LPA, and 1.9h of MVPA (Dumuid, 2022) | Dumuid 2022 |
|  |  |  | Isotemporal Substitution |  |  |
|  |  |  |  | When not meeting the SL guideline: -1/1 studies showed replacing ST with MVPA or SL was associated with more favorable scores (Gilchrist, 2021) -1/1 studies showed replacing SL with MVPA was associated with less favorable scores (Gilchrist, 2021)  When meeting the SL guideline: -1/1 studies showed that replacing SL with ST was associated with less favorable scores -1/1 studies showed that replacing ST with MVPA or SL was associated with more favorable scores | Gilchrist 2021 |
|  |  |  | Latent Profiles |  |  |
|  |  |  |  | 2/2 studies found the healthiest profiles/clusters had the most favorable scores | Cao 2020, Brown 2021 |
|  |  | Quality of Life |  |  |  |
|  |  |  | Total Guidelines |  |  |
|  |  |  |  | 1/1 studies found a favorable dose-response relationship for number of guidelines met | Li 2022 |
|  |  |  | Combinations of Guidelines |  |  |
|  |  |  |  | 1/1 studies showed that compared to meeting all 3 guidelines, meeting PA+SL, PA+ST, SL+ST were associated with the most favorable scores (no differences) | Li 2022 |
|  |  |  | Goldilocks Method |  |  |
|  |  |  |  | 1/1 studies found the optimal 24-hr composition was 9.7h of SL, 10.5h of SB, 1.7h of LPA, and 2.1h of MVPA | Dumuid 2022 |
|  |  | Self Esteem |  |  |  |
|  |  |  | Guidelines |  |  |
|  |  |  |  | 1/1 studies found a favorable dose-response relationship for number of guidelines met | Sampasa-Kanyinga 2022 |
|  |  |  | Combinations of Guidelines |  |  |
|  |  |  |  | 1/1 studies showed that compared to meeting none of the guidelins, meeting all 3 guidelines or the SL+ST guidelines were associated with the most favorable scores | Sampasa-Kanyinga 2022 FiPH |
|  |  |  | Compositional |  |  |
|  |  |  |  | 0/2 studies found an association with the 24-h movement composition | Fairclough 2021 |
|  |  |  | Isotemporal Substitution |  |  |
|  |  |  |  | 1/1 studies showed that replacing ST with MVPA or SL was associated with more favorable scores | Brown 2021 FBN |
|  |  |  | Latent profiles |  |  |
|  |  |  |  | 1/1 study showed the the healthiest combination of movement behaviors (adequate SL, high MVPA, low ST) was associated with more favorable scores | Brown 2021 MENPA |
|  |  | Resilience |  |  |  |
|  |  |  | Isotemporal Substitution |  |  |
|  |  |  |  | 1/1 studies showed that replacing ST with MVPA or SL was associated with more favorable scores | Brown 2021 FBN |
|  |  |  | Latent Profiles |  |  |
|  |  |  |  | 1/1 study showed the the healthiest combination of movement behaviors (adequate SL, high MVPA, low ST) was associated with more favorable scores | Brown 2021 MENPA |
|  |  |  | Total Guidelines |  |  |
|  |  |  |  | 1/1 studies showed that meeting all 3 guidelines was associated with the most favorable scores compared to meeting none of the guidelines | Liang 2023 |
|  |  |  | Combinations of Guidelines |  |  |
|  |  |  |  | 1/2 studies showed that meeting all 3 guidelines was associated with the most favorable scores compared to meeting none of the guidelines  ½ studies showed that meeting the SL and PA guidelines was associated with the most favorable score | Liang 2023; Hou 2023 |
|  |  |  | Compositional |  |  |
|  |  |  |  | 1/1 studies found that relative to other behaviors, a favorable association was observed for MVPA with resilience | Taylor 2023 |
|  |  | Prosocial Behavior |  |  |  |
|  |  |  | Total Guidelines |  |  |
|  |  |  |  | 1/2 studies showed that meeting all 3 guidelines was associated with the most favorable scores compared to meeting none of the guidelines (Janssen 2017) | McNeill 2020; Janssen 2017 |
|  |  |  | Combinations of Guidelines |  |  |
|  |  |  |  | 1/5 studies showed that meeting all 3 guidelines was associated with the most favorable scores compared to meeting none of the guidelines (Janssen, 2017) | Christian 2022, Janssen 2017, McNeill 2020; Kuzik 2022 |
|  |  |  | Compositional |  |  |
|  |  |  |  | 1/4 studies found an association with the 24-h movement composition among primary school children, and SB was associated with less favorable scores (Fairclough 2021) | Kuzik 2020; fairclough 2021; chong 2021 |
|  |  |  | Isotemporal Substitution |  |  |
|  |  |  |  | 1/2 studies showed that replacing SB with SL, LPA or MVPA was associated with more favorable scores among primary school children (Fairclough, 2021) | Fairclough 2021 |
|  |  | Life Satisfaction |  |  |  |
|  |  |  | Total Guidelines |  |  |
|  |  |  |  | 2/2 studies found a favorable dose-response relationship for number of guidelines met | Janssen 2017, Peralta 2022 |
|  |  |  | Combinations of Guidelines |  |  |
|  |  |  |  | 1/2 studies showed that meeting all 3 guidelines was associated with more favorable scores compared to all other combinations  1/2 studies showed that compared to meeting one guideline, meeting the PA + ST was associated with the most favorable scores | Janssen 2017, Peralta 2022 |
|  |  |  | Goldilocks Method |  |  |
|  |  |  |  | 1/1 studies found the optimal 24-hr composition was 11.4h of SL; 7.5h of SB; 2.9h of LPA; 2.2h of MVPA | Dumuid 2022 |
|  |  | Psychological Distress |  |  |  |
|  |  |  | Compositional |  |  |
|  |  |  |  | 1/2 studies found an association with the 24-h movement composition, which was driven by a postitive association with SB and a negative association with LPA | Chong 2021, Faria (2022) |
|  |  |  | Isotemporal Subsitution |  |  |
|  |  |  |  | 1/1 studies showed that replacing SB with LPA was associated with more favorable scores  1/1 studies showed that replacing LPA with SB was associated with less favourable scores 1/1 studies showed that replacing MVPA with LPA was associated with more favorable scores | Faria (2022) |
|  |  | Mental Health |  |  |  |
|  |  |  | Total Guidelines |  |  |
|  |  |  |  | 2/3 studies found a favorable dose-response relationship for number of guidelines met (Sampasa-Kanyinga 2022) | Sampasa-Kanyinga 2022 CJoPH, Bang 2020 |
|  |  |  | Combinations of Guidelines |  |  |
|  |  |  |  | 1/3 studies found that compared to not meeting the guidelines, meeting the PA+SL guidelines was associated with the most favorable scores (Sampasa-Kanyinga, 2022; 2017 cycle) 1/3 studies found that compared to not meeting the guidelines, meeting all 3 guidelines was associated with the most favorable scores (Sampasa-Kanyinga, 2022; 2019 cycle) | Bang 2020, Sampasa-Kanyinga 2022 |
|  |  | Loneliness |  |  |  |
|  |  |  | Combinations of Guidelines |  |  |
|  |  |  |  | 1/1 studies showed that compared to meeting none of the guidelines, meeting the PA + SL guidelines was associated with the most favorable scores | Burns 2020 |
|  |  | Prolonged Sadness |  |  |  |
|  |  |  | Combinations of Guidelines |  |  |
|  |  |  |  | 1/1 study showed that compared to meeting none of the guidelines, meeting all 3 guidelines was associated with the most favorable scores | Burns 2020 |
|  |  | Suicidal Ideation |  |  |  |
|  |  |  | Total Guidelines |  |  |
|  |  |  |  | 2/4 studies showed that compared to meeting none of the guidelines, meeting all 3 guidelines was associated with the most favorable scores among boys but not girls (Liu, 2022; Sampasa-Kanyinga 2020) | Liu 2022, Sampasa-Kanyinga 2020 |
|  |  |  | Combinations of Guidelines |  |  |
|  |  |  |  | 1/4 studies showed that compared to meeting none of the guidelines, meeting the PA+ST guidelines was associated with the most favorable scores for girls aged 11-14 years (Sampasa-Kanyinga 2020) 1/4 studies showed that compared to meeting none of the guidelines, meeting the ST+SL guidelines was associated with the most favorable scores for girls aged 15-20 years (Sampasa-Kanyinga 2020) 1/4 studies showed that compared to meeting none of the guidelines, meeting all 3 guidelines was associated with the most favorable scores for 15-20 year old boys (Sampasa-Kanyinga 2020) | Sampasa-Kanyinga 2020 |
|  |  | Suicidal Planning |  |  |  |
|  |  |  | Total Guidelines |  |  |
|  |  |  |  | 2/4 studies showed that compared to meeting none of the guidelines, meeting all 3 guidelines was associated with the most favorable scores among boys but not girls (Liu, 2022; Sampasa-Kanyinga 2020) | Liu 2022, Sampasa-Kanyinga 2020 |
|  |  | Suicide (attempts) |  |  |  |
|  |  |  | Total Guidelines |  |  |
|  |  |  |  | 1/4 studies showed that compared to meeting none of the guidelines, meeting all 3 guidelines was associated with the most favorable scores among boys (Sampasa-Kanyinga 2020) | Liu 2022, Sampasa-Kanyinga 2020 |
|  |  |  | Combinations of Guidelines |  |  |
|  |  |  |  | 1/4 studies showed that compared to meeting none of the guidelines, meeting the ST guideline was associated with the most favorable scores for girls aged 11-14 years (Sampasa-Kanyinga 2020) 1/4 studies showed that compared to meeting none of the guidelines, meeting the PA guideline was associated with the most favorable scores for girls aged 15-20 years (Sampasa-Kanyinga 2020) 1/4 studies showed that compared to meeting none of the guidelines, meeting all 3 guidelines was associated with the most favorable scores for 15-20 year old boys (Sampasa-Kanyinga 2020) | Sampasa-Kanyinga 2020 |
|  |  | Anxiety |  |  |  |
|  |  |  | Total Guidelines |  |  |
|  |  |  |  | 1/9 studies found a favorable dose-response relationship for number of guidelines met (Liang, 2023) 4/9 studies showed that compared to meeting none of the guidelines, meeting all 3 guidelines was associated with the most favorable scores (Feng 2022; Lu 2021; Luo 2023; Zhang 2023) 2/9 studies showed that compared to meeting none of the guidelines, meeting 2 guidelines was associated with the most favorable scores (Brown 2021 JPAH; Feng 2022)  3/9 studies showed no differences between meeting any number of guidelines compared to meeting none of the guidelines (Brown, 2021 EB) | Feng 2022; Brown 2021; Brown 2021; Lu 2021, Liang 2023; Luo 2023; Zhang 2023 |
|  |  |  | Combinations of Guidelines |  |  |
|  |  |  |  | 3/8 studies showed that compared to meeting none of the guidelines, meeting all 3 guidelines was associated with the most favorable scores (Lu, 2021; Zhu, 2019 12-17 year old sample, Zhang 2023) 4/8 studies showed that compared to meeting none of the guidelines, meeting the ST + SL guidelines was associated with the most favorable scores (Feng, 2021; Liang 2023; Sampasa-Kanyinga, 2021; Hou 2023) | Lu 2021; Sampasa-Kaniynga (2021); Feng 2022; Zhu 2019; Liang 2023; Hou 2023, Zhang 2023 |
|  |  |  | Isotemporal Substitution |  |  |
|  |  |  |  | When not meeting the SL guideline: -1/1 studies showed that replacing ST or MVPA with SL was associated with more favorable scores  When meeting the SL guideline: 1/1 studies showed that replacing ST with MVPA or SL was associated with more favorable scores | Gilchrist 2021 |
|  |  |  | Compositional |  |  |
|  |  |  |  | 1/1 studies found that relative to other behaviors, a favorable association was observed for MVPA with anxiety | Taylor 2023 |
|  |  | Emotional Problems |  |  |  |
|  |  |  | Total Guidelines |  |  |
|  |  |  |  | 1/3 studies showed that compared to meeting none of the guidelines, meeting all 3 guidelines was associated with the most favorable scores (Janssen, 2017) | Janssen 2017, Christian 2022 |
|  |  |  | Combinations of Guidelines |  |  |
|  |  |  |  | 1/3 studies showed that compared to meeting none of the guidelines, meeting all 3 guidelines was associated with the most favorable scores (Janssen, 2017) | Janssen 2017, Christian 2022 |
|  |  | Emotional, Behavioral, and Social Problems (total difficulties) |  |  |  |
|  |  |  | Total Guidelines |  |  |
|  |  |  |  | 1/6 studies showed that compared to meeting none of the guidelines, meeting 2+ guidelines was associated with the most favorable scores among youth (Bang 2020) 3/6 studies showed that compared to meeting none of the guidelines, meeting all 3 guidelines was associated with the most favorable scores (Carson, 2019; Lopez-Gil, 2022) | Lopez-Gil 2022; Bang 2020; Carson 2019, McNeil(2020) |
|  |  |  | Combinations of Guidelines |  |  |
|  |  |  |  | 1/5 studies showed that compared to meeting none of the guidelines, meeting the SL+PA guidelines was associated with the most favorable scores among youth (Bang 2020) 1/5 studies showed that compared to meeting none of the guidelines, meeting the ST guideline was associated with the most favorable scores among boys (Christian, 2022) | Christian 2022, Bang 2020; McNeill 2020 |
|  |  |  | Compositional Analysis |  |  |
|  |  |  |  | 3/4 showed an association with the 24-h movement composition (Chong 2021, Dumuid), with this relationship driven by a negative association for SL (Chong 2021, Fairclough 2023) and positive associations for LPA (Chong 2021) and ST (Chong 2021; Fairclough 2023). | Chong 2021, Dumuid 2022, Fairclough 2021; Fairclough 2023 |
|  |  |  | Goldilocks Methods |  |  |
|  |  |  |  | 1/1 studies found the optimal 24-hr composition was 11.4h of SL, 7.3h of SB, 3.0h of LPA, 2.2h of MVPA (Dumuid 2022)  1/1 studies found the optimal 24-hour composition was  SL = 10h, ST = 6.5h, LPA = 6.9 hours, and MPA and VPA = 43 min (Fairclough 2023) | Dumuid 2022; Fairclough 2023 |
|  |  | Rest-activity Rhythmicity |  |  |  |
|  |  |  |  | 1/1 associations showed a significant negative relationship (more stability) was observed between inter-daily stability and total difficulties (Fairlough, 2023)  0/1 associations demonstrated a significant relationship for intra-day variability with indicators of mental health | Fairlough, 2023 |
|  |  | Internalising Problems |  |  |  |
|  |  |  | Total Guidelines |  |  |
|  |  |  |  | 1/2 studies found a favorable dose-response relationship for number of guidelines met (Carson 2019)  ½ studies showed that meeting none of the guidelines, PA + SL, or PA + ST had higher odds of internalizing problems compared to meeting all three guidelines. (Zhu 2023) | Carson 2019; McNeill 2020; Zhu 2023 |
|  |  |  | Combinations of Guidelines |  |  |
|  |  |  |  | 1/3 studies showed that compared to not meeting the guidelines, meeting all 3 guidelines was associated with the most favorable scores (Sampasa-Kanyinga 2021) | McNeill 2020, Sampasa-Kanyinga 2021; Kuzik 2022 |
|  |  |  | Compositional Analysis |  |  |
|  |  |  |  | 1/6 studies found an association with the 24-h movement composition (Chong 2021), which was driven by SB, SL and LPA (Chong 2021) | Kuzik 2020,Fairclough 2021; Chong 2021; Fairclough 2023; St Laurent 2023 |
|  |  |  | Isotemporal Substitution |  |  |
|  |  |  |  | 1/1 studies showed that replacing SB with MVPA was associated with more favorable scores (Kuzik 2020) 1/1 studies showed that replacing SL with MVPA was associated with more favorable scores (Kuzik 2020) | Kuzik 2020; Fairclough 2021 |
|  |  |  | Rest-activity Rhythmicity |  |  |
|  |  |  |  | 0/1 associations showed a significant negative relationship (more stability) was observed between inter-daily stability and internalizing behaviors  0/1 associations demonstrated a significant relationship for intra-day variability with indicators of mental health | Fairlough, 2023 |
|  |  | Sociability |  |  |  |
|  |  |  | Compositional Analysis |  |  |
|  |  |  |  | 0/1 studies found an association with the 24-h movement composition | Kuzik 2020 |
|  |  |  | Isotemporal Substitution |  |  |
|  |  |  |  | 1/1 studies showed that replacing SB, LPA or SL with MVPA was associated with more favorable scores | Kuzik 2020 |
|  |  |  | Combination Guidelines |  |  |
|  |  |  |  | 0/1 found an association with combinations of guidelines | Kuzik 2022 |
|  |  | Peer Problems |  |  |  |
|  |  |  | Combinations of Guidelines |  |  |
|  |  |  |  | 0/2 found an association with combinations of guidelines | Christian 2022 |
|  |  | Happiness |  |  |  |
|  |  |  | Total Guidelines |  |  |
|  |  |  |  | 1/1 studies showed that compared to meeting none of the guidelines, meeting all 3 guidelines was associated with the most favorable scores | Lee 2018 |
|  |  |  | Combinations of Guidelines |  |  |
|  |  |  |  | 1/1 studies showed that compared to not meeting the guidelines, meeting the PA+ST guidelines was associated with the most favorable scores | Lee 2018 |
|  |  | Well-being |  |  |  |
|  |  |  | Total Guidelines |  |  |
|  |  |  |  | 2/2 studies showed that compared to meeting none of the guidelines, meeting all 3 guidelines was associated with the most favorable scores | Liang 2023; Sun 2023 |
|  |  |  | Combinations of Guidelines |  |  |
|  |  |  |  | 1/2 studies showed that compared to meeting none of the guidelines, meeting all 3 guidelines was associated with the most favorable scores  1/2 studies showed that compared to meeting none of the guidelines, meeting PA+SL guidelines was associated with the most favorable scores (sun 2023) | Liang 2023; sun 2023 |
|  | Longitudinal |  |  |  |  |
|  |  | Internalizing Problems |  |  |  |
|  |  |  | Compositional Analysis |  |  |
|  |  |  |  | 0/1 studies found an association with the 24-h movement composition | Chong 2021 |
|  |  |  | Total Guidelines |  |  |
|  |  |  |  | 0/1 studies showed that meeting any number of guidelines was associated with more favorable scores compared to meeting none of the guidelines | McNeill 2020 |
|  |  |  | Combinations of Guidelines |  |  |
|  |  |  |  | 1/2 studies showed that compared to meeting none of the guidelines at both time poitns, meeting the ST+PA guidelines was associated with the most favorable change scores (Fung, 2022) | Fung 2022, McNeill 2020 |
|  |  | Emotional/ Behavioural/ Social difficulties (total difficulties) |  |  |  |
|  |  |  | Compositional Analysis |  |  |
|  |  |  |  | 0/1 studies found an association with the 24-h movement composition | Chong 2021 |
|  |  |  | Total Guidelines |  |  |
|  |  |  |  | 0/2 studies showed that meeting any number of guidelines was associated with more favorable scores compared to meeting none of the guidelines | McNeill 2020, Hinkley 2020 |
|  |  |  | Combinations Guidelines |  |  |
|  |  |  |  | 1/2 studies showed that compared to meeting none of the guidelines at both time points, meeting the SL+ST guidelines was associated with more favorable change scores (Fung, 2022) | McNeill 2020, Fung 2022 |
|  |  | Prosocial Behaviour |  |  |  |
|  |  |  | Compositional Analysis |  |  |
|  |  |  |  | 0/1 studies found an association with the 24-h movement composition | Chong 2021 |
|  |  |  | Total Guidelines |  |  |
|  |  |  |  | 0/2 studies showed that meeting any number of guidelines was associated with more favorable scores compared to meeting none of the guidelines | McNeill 2020, Hinkley 2020 |
|  |  |  | Combinations Guidelines |  |  |
|  |  |  |  | 0/1 studies showed that meeting any combination of guidelines was associated with more favorable scores compared to meeting none of the guidelines | McNeill 2020 |
|  |  | Psychological Distress |  |  |  |
|  |  |  | Compositional Analysis |  |  |
|  |  |  |  | 0/1 studies found an association with the 24-h movement composition | Chong 2021 |
|  |  | Depression |  |  |  |
|  |  |  | Total Guidelines |  |  |
|  |  |  |  | 1/4 studies showed that compared to meeting none of the guidelines, meeting all 3 guidelines was associated with more favorable scores at age 5 but not age 1 or 2 (Taylor 2021)  1/4 studies showed meeting all three guidelines was associated with the most favorable scores compared to meeting none of the guidelines 6 months later (Zhang, 2023) | Taylor 2021; Zhang 2023 |
|  |  |  | Combination Guidelines |  |  |
|  |  |  |  | 1/1 study showed meeting all three guidelines was associated with the most favorable scores compared to meeting none of the guidelines 6 months later (Zhang, 2023) | Zhang, 2023) |
|  |  |  | Compositional Analysis |  |  |
|  |  |  |  | 0/6 studies found an association with the 24-h movement composition  4/6 studies showed that relative to other behaviors, SL and ST were associated with more favorable scores among younger and older boys and girls 1/6 studies showed that relative to other behaviors, MVPA was associated with more favorable scores among older girls only | Sampasa-Kanyinga 2021; Taylor 2023 |
|  |  |  | Isotemporal Substitution |  |  |
|  |  |  |  | 5/5 studies showed that replacing ST with MVPA or SL was associated with more favorable scores among younger and older boys and girls (Sampasa-Kanyinga 2021) and adolescents (Duncan 2022) 4/5 studies showed that replacing MVPA with SL was associated with more favorable scores among younger and older boys as well as younger girls (Sampasa-Kanyinga 2021) and adolescents (Duncan 2022) | Sampasa-Kanyinga 2021, Duncan 2022 |
|  |  |  | Latent Profiles |  |  |
|  |  |  |  | 1/1 studies showed the the healthiest combination of movement behaviors (adequate sleep, high MVPA, low ST) was associated with the most favorable scores | Brown 2021 PM |
|  |  | Anxiety |  |  |  |
|  |  |  | Total Guidelines |  |  |
|  |  |  |  | 1/3 studies showed that compared to meeting none of the guidelines, meeting all 3 guidelines was associated with more favorable scores at age 1 but not age 2 or 5 (Taylor 2021)  1/1 study showed meeting all three guidelines was associated with the most favorable scores compared to meeting none of the guidelines 6 months later (Zhang, 2023) | Taylor 2021; Zhang 2023 |
|  |  |  | Isotemporal Substitution |  |  |
|  |  |  |  | 1/1 studies showed that for between-person associations, replacing SL or ST with MVPA was associated with more favorable scores | Duncan 2022 |
|  |  |  | Combination Guidelines |  |  |
|  |  |  |  | 1/1 study showed meeting all three guidelines was associated with the most favorable scores compared to meeting none of the guidelines 6 months later (Zhang, 2023) | Zhang 2023 |
|  |  |  | Compositional |  |  |
|  |  |  |  | 0/1studies found an association with the 24-h movement composition | Taylor 2023 |
|  |  | Resilience |  |  |  |
|  |  |  | Total Guidelines |  |  |
|  |  |  |  | 0/3 studies showed that meeting any number of guidelines was associated with more favorable scores compared to meeting none of the guidelines at ages 1, 2 or 5 | Taylor 2021 |
|  |  |  | Compositional |  |  |
|  |  |  |  | 0/1 studies found an association with the 24-h movement composition | Taylor 2023 |
|  |  | Quality of Life - Psychosocial |  |  |  |
|  |  |  | Total Guidelines |  |  |
|  |  |  |  | 0/1 studies showed that meeting any number of guidelines was associated with more favorable scores compared to meeting none of the guidelines | Hinkley 2020 |
|  |  | Self-esteem/ Self-worth |  |  |  |
|  |  |  | Total Guidelines |  |  |
|  |  |  |  | 0/1 studies showed that meeting any number of guidelines was associated with more favorable scores compared to meeting none of the guidelines | Hinkley 2020 |
|  |  | Flourishing |  |  |  |
|  |  |  | Isotemporal Substitution |  |  |
|  |  |  |  | 1/1 showed that for between-person associations, replacing ST with MVPA was associated with more favorable scores  1/1 showed that for between-person associations, replacing ST with SL was associated with more favorable scores | Duncan 2022 |
|  |  | Emotional Dysregulation |  |  |  |
|  |  |  | Isotemporal Subsitution |  |  |
|  |  |  |  | 1/1 studies showed that for between-person associations, replacing ST with MVPA or SL was associated with more favorable scores 1/1 studies showed that for between-person associations, replacing MVPA with SL was associated with more favorable scores | Duncan 2022 |
| Adults |  |  |  |  |  |
|  | Cross-Sectional |  |  |  |  |
|  |  | Depression |  |  |  |
|  |  |  | Compositional |  |  |
|  |  |  |  | 1/3 studies showed that the baseline 24-hr movement composition was prospectively associated with more favorable scores (Cabanas-Sanchez 2021) 2/5 studies showed that time spent in MVPA (relative to other behaviors) was associated with more favorable scores (Blodgett 2023, Cabanas-Sanchez 2021)  1/5 studies showed that time spent in LPA (relative to other behaviors) was associated with more favorable scores (Blodgett 2023) 1/5 studies showed that time spent in SL (relative to other behaviors) was associated with less favorable scores (Blodgett 2023) 1/5 studies showed that time spent in SB (relative to other behaviors) was associated with less favorable scores (delPozoCruz 2020) | Curtis 2020; Larisch 2020; delPozoCruz 2020; Blodgett 2023; Cabanas-Sanchez 2021 |
|  |  |  | Total Guidelines |  |  |
|  |  |  |  | 1/1 studies showed that compared to meeting none of the guidelines, meeting all three was associated with the most favorable scores | Ohta 2023 |
|  |  |  | Combinations of Guidelines |  |  |
|  |  |  |  | 2/2 studies showed that compared with meeting none of the guidelines, meeting all 3 guidelines was associated with the most favorable scores | Liang 2021; Ohta 2023 |
|  |  |  | Isotemporal Substituion |  |  |
|  |  |  |  | 4/5 studies showed that replacing SB with MVPA was associated with more favorable scores 3/5 studies showed that replacing SL with MVPA was associated with more favorable scores (Cabanas-Sanchez 2021; Hofman, 2022; Blodgett 2023)  2/5 studies showed that replacing LPA with MVPA was associated with more favorable scores (Cabanas-Sanchez 2021, Blodgett 2023)  1/5 studies showed that replacing SB with SL was associated with more favorable scores (Hofman, 2022) 1/5 studies showed that replacing LPA with SL or SB was associated with more favorable scores (Cabanas-Sanchez 2021) 2/5 studies showed that replacing SL or SB with LPA was associated with more favorable scores (Blodgett 2023, Curtis 2023) | Hofman 2022; delPozoCruz 2020; Cabanas-Sanchez 2021; Blodgett 2023; Curtis 2023 |
|  |  | Anxiety |  |  |  |
|  |  |  | Compositional |  |  |
|  |  |  |  | 1/3 studies found an association with the 24-h movement composition (Chao 2022) | Curtis 2020; Larisch 2020; Chao 2022 |
|  |  |  | Total Guidelines |  |  |
|  |  |  |  | 1/1 studies found a favorable dose-response relationship for number of guidelines met | Bu 2021 |
|  |  |  | Combinations of Guidelines |  |  |
|  |  |  |  | 1/1 showed that compared to meeting none of the guidelines, meeting the SL + MVPA guidelines was associated with the most favorable scores | Liang 2021 |
|  |  |  | Isotemporal Substituion |  |  |
|  |  |  |  | 1/3 studies showed that replacing LPA with SB, SL or MPVA was associated with more favorable scores (Zhang 2022) 1/3 studies showed that replacing MVPA or SB with SL was associated with more favorable scores (Zhang 2022)  1/3 studies showed that replacing SL or SB with LPA was associated with more favorable scores (Curtis 2023) | Hofman 2022; Zhang 2022; Curits 2023 |
|  |  | Psychological Distress |  |  |  |
|  |  |  | Compositional |  |  |
|  |  |  |  | 1/2 studies found an association with the 24-h movement composition during the work day, but not for non-work days (Kitano 2020), which was driven by favorable associations with SL and unfavorable associations for SB and LPA | Kitano 2020 |
|  |  |  | Total Guidelines |  |  |
|  |  |  |  | 1/3 studies showed that compared to meeting none of the guidelines, meeting some of the guidelines 2/3 studies showed that compared to meeting none of the guidelines, meeting all 3 guidelines was associated with more favorable scores among men | Perez 2022; Porter 2023 |
|  |  |  | Profiles |  |  |
|  |  |  |  | 1/1 studies showed that one of the two healthiest combinations of movement behaviors (adequate sleep, high MVPA, low ST) was associated with the most favorable scores | Brown 2022 |
|  |  | General Mental Health |  |  |  |
|  |  |  | Compositional |  |  |
|  |  |  |  | 0/1 studies found an association with the 24-h movement composition | Cabanas-Sanchez 2021 |
|  |  |  | Isotemporal Substituion |  |  |
|  |  |  |  | 1/2 studies showed that replacing SB with LPA or SL was associated with more favorable scores | Colley 2018, Cabanas-Sanchez 2021 |
|  |  |  | Total Guidelines |  |  |
|  |  |  |  | 1/6 studies showed that compared to meeting none of the guidelines, meeting all 3 guidelines was assocaited with the most favorable scores among females (Luo 2022) 1/6 studies showed that compared to meeting none of the guidelines, meeting 1 guideline was associated with the most favorable scores among individuals with Class 1 Obesity (Baillot, 2022) | Baillot 2022, Luo, 2022 |
|  |  |  | Combination of Guidelines |  |  |
|  |  |  |  | 1/6 studies showed that meeting the SL guideline was associated with more favorable scores among individuals with Class 1 Obesity (Baillot, 2022) 1/6 studies showed that compared to meeting none of the guidelines, meeting the PA+SL guidelines was associated with more favorable scores among men (Luo, 2022) 1/6 studies showed that compared to meeting none of the guidelines, meeting all 3 guidelines guidelines was associated with more favorable scores among women (Luo, 2022) | Baillot 2022, Luo, 2022 |
|  |  | Mood States |  |  |  |
|  |  |  | Latent Profiles |  |  |
|  |  |  |  | 1/1 studies showed the the healthiest combination of movement behaviors (adequate sleep, high MVPA, low SB) was associated with the most favorable scores | Hajo 2020 |
|  |  |  | Isotemporal Substitution |  |  |
|  |  |  |  | 1/1 studies showed that replacing SB with SL was associated with more favorable scores | Meyer 2020 |
|  |  | Well-being |  |  |  |
|  |  |  | Compositional |  |  |
|  |  |  |  | 0/2 studies found an association with the 24-h movement composition | Larisch 2020; Vanderlinden 2023 |
|  |  |  | Isotemportal Substitution | 0/1 studies found an association | Vanderlinden 2023 |
|  |  |  | Total Guidelines | 1/1 studies showed that compared to meeting none of the guidelines, meeting all 3 guidelines was associated with more favorable scores among men | Porter 2023 |
|  |  |  |  |  |  |
|  |  |  | Profiles |  |  |
|  |  |  |  | 1/1 studies showed that the two healthiest combinations of movement behaviors (adequate sleep, high or very high MVPA, low ST) were associated with the most favorable scores | Brown 2022 |
|  |  | PTSD |  |  |  |
|  |  |  | Total Guidelines |  |  |
|  |  |  |  | 1/2 studies showed that compared to meeting none of the guidelines, meeting all 3 guidelines was associated with the most favorable scores among men, but not women (Perez 2022) | Perez 2022 |
|  |  | Suicide Ideation |  |  |  |
|  |  |  | Total Guidelines |  |  |
|  |  |  |  | 2/2 studies showed that compared to meeting none the guidelines, meeting all 3 guidelines was associated with the most favorable scores for men and women | Perez 2022 |
|  |  |  | Combinations of Guidelines |  |  |
|  |  |  |  | 1/1 studies showed that compared to meeting none of the guidelines, meeting all 3 guidelines was associated with the most favorable scores | Brown 2022 |
|  |  | Suicide planning |  |  |  |
|  |  |  | Combinations of Guidelines |  |  |
|  |  |  |  | 1/1 studies showed that compared to meeting none of the guidelines, meeting the PA+SL guidelines was associated with the most favorable scores | Brown 2022 |
|  |  | Loneliness |  |  |  |
|  |  |  | Compositional |  |  |
|  |  |  |  | 0/1 studies found an association with the 24-h movement composition, although relative to other behaviors, a favorable association was observed for MVPA | Cabanas-Sánchez 2021 |
|  |  |  | Isotemporal Substituion |  |  |
|  |  |  |  | 1/1 studies showed that replacing SB, LPA or SL with MVPA was associated with more favorable scores | Cabanas-Sánchez 2021 |
|  |  | Happiness |  |  |  |
|  |  |  | Compositional |  |  |
|  |  |  |  | 1/1 studies found a signficant association with the 24-h movement composition; relative to other behaviors, a favorable association was observed for MVPA | Cabanas-Sánchez 2021 |
|  |  |  | Isotemporal Substituion |  |  |
|  |  |  |  | 1/1 studies showed that replacing SB, LPA or SL with MVPA was associated with more favorable scores | Cabanas-Sánchez 2021 |
|  | Longitudinal |  |  |  |  |
|  |  | Daily Affect |  |  |  |
|  |  |  | Isotemporal Substituion |  |  |
|  |  |  |  | 1/4 studies showed that replacing SL, SB or LPA with MVPA was associated with more favorable scores (high arousal positive affect only) | Le 2021 |
|  |  | Mood States |  |  |  |
|  |  |  | Isotemporal Substituion |  |  |
|  |  |  |  | 1/1 studies showed that replacing prolonged SB with LPA or MVPA was associated with more favorable scores | Meyer 2020 |
|  |  | Depression |  |  |  |
|  |  |  | Total Guidelines |  |  |
|  |  |  |  | 1/1 studies showed that compared to meeting none of the guidelines in adolescence and adulthood, meeting all 3 guidelines was associated with more favorable scores in adulthood (Garcia-Hermoso 2022) | Garcia-Hermoso, 2022 |
|  |  |  | Isotemporal Substituion |  |  |
|  |  |  |  | 1/2 studies showed that replacing SB with LPA, MVPA or SL was associated with more favorable scores (Kandola 2021) 1/2 studies showed that replacing SL with SB was associated with more favorable scores (Cabanas-Sanchez 2021) | Kandola 2021; Cabanas-Sanchez 2021 |
|  |  |  | Compositional |  |  |
|  |  |  |  | 0/1 studies found an association with the 24-h movement composition; although relative to the other behaviors, an unfavorable association was observed for SL | Cabanas-Sanchez 2021 |
|  |  | Anxiety |  |  |  |
|  |  |  | Isotemporal Substituion |  |  |
|  |  |  |  | 1/1 studies showed that replacing LPA with SB was associated with more favorable scores  1/1 studies showed that replacing SB with SL or MVPA was associated with more favorable scores | Kandola 2021 |
|  |  | Loneliness |  |  |  |
|  |  |  | Compositional |  |  |
|  |  |  |  | 0/1 studies found an association with the 24-h movement composition | Cabanas-Sanchez 2021 |
|  |  |  | Isotemporal Substitution |  |  |
|  |  |  |  | 0/1 studies found effects of reallocating time across movement behaviors | Cabanas-Sanchez 2021 |
|  |  | Happiness |  |  |  |
|  |  |  | Compositional |  |  |
|  |  |  |  | 0/1 studies found an association with the 24-h movement composition | Cabanas-Sanchez 2021 |
|  |  |  | Isotemporal Substituion |  |  |
|  |  |  |  | 0/1 studies found effects of reallocating time across movement behaviors | Cabanas-Sanchez 2021 |
|  |  | Mental Health |  |  |  |
|  |  |  | Compositional |  |  |
|  |  |  |  | 0/1 studies found an association with the 24-h movement composition; although relative to the other behaviors, a favorable association was observed for MVPA | Cabanas-Sanchez 2021 |
|  |  |  | Isotemporal Substitution |  |  |
|  |  |  |  | 1/1 studies showed that replacing SB, LPA or SL with MVPA was associated with more favorable scores | Cabanas-Sanchez 2021 |
|  |  | Suicidal Ideation |  |  |  |
|  |  |  | Total Guidelines |  |  |
|  |  |  |  | 1/1 studies showed that compared to meeting none of the guidelines in adolescence and adulthood, meeting all 3 guidelines was associated with more favorable scores in adulthood | Garcia-Hermoso 2022 |
